# Engineered proteins detect spontaneous DNA breakage in human and bacterial cells

**DOI:** 10.7554/eLife.01222

**Published:** 2013-10-29

**Authors:** Chandan Shee, Ben D Cox, Franklin Gu, Elizabeth M Luengas, Mohan C Joshi, Li-Ya Chiu, David Magnan, Jennifer A Halliday, Ryan L Frisch, Janet L Gibson, Ralf Bernd Nehring, Huong G Do, Marcos Hernandez, Lei Li, Christophe Herman, PJ Hastings, David Bates, Reuben S Harris, Kyle M Miller, Susan M Rosenberg

**Affiliations:** 1 Department of Molecular and Human Genetics, Baylor College of Medicine, Houston, United States; 2 Department of Molecular Virology and Microbiology, Baylor College of Medicine, Houston, United States; 3 Dan L Duncan Cancer Center, Baylor College of Medicine, Houston, United States; 4 Department of Biochemistry, Molecular Biology, Baylor College of Medicine, Houston, United States; 5 Institute for Cellular and Molecular Biology, and Department of Molecular Biosciences, University of Texas, Austin, United States; 6 Department of Biochemistry, Molecular Biology and Biophysics, Masonic Cancer Center, University of Minnesota, Minneapolis, United States; 7 Department of Experimental Radiation Oncology, the University of Texas MD Anderson Cancer Center, Houston, United States; Harvard Medical School, United States

**Keywords:** DNA double-strand breaks, endogenous DNA damage, GFP, fluorescent-protein fusions, spontaneous DNA breaks, synthetic biology, *E. coli*, Human, Mouse

## Abstract

Spontaneous DNA breaks instigate genomic changes that fuel cancer and evolution, yet direct quantification of double-strand breaks (DSBs) has been limited. Predominant sources of spontaneous DSBs remain elusive. We report synthetic technology for quantifying DSBs using fluorescent-protein fusions of double-strand DNA end-binding protein, Gam of bacteriophage Mu. In *Escherichia coli* GamGFP forms foci at chromosomal DSBs and pinpoints their subgenomic locations. Spontaneous DSBs occur mostly one per cell, and correspond with generations, supporting replicative models for spontaneous breakage, and providing the first true breakage rates. In mammalian cells GamGFP—labels laser-induced DSBs antagonized by end-binding protein Ku; co-localizes incompletely with DSB marker 53BP1 suggesting superior DSB-specificity; blocks resection; and demonstrates DNA breakage via APOBEC3A cytosine deaminase. We demonstrate directly that some spontaneous DSBs occur outside of S phase. The data illuminate spontaneous DNA breakage in *E. coli* and human cells and illustrate the versatility of fluorescent-Gam for interrogation of DSBs in living cells.

**DOI:** http://dx.doi.org/10.7554/eLife.01222.001

## eLife digest

Cells have developed a variety of mechanisms for repairing DNA molecules when breaks occur in one or both of the DNA strands. However, we know relatively little about the causes of these breaks, which often occur naturally, or even about how common they are. Learning more about the most common forms of DNA breakage is important because the genomic changes caused by these breaks are driving forces behind both cancer and evolution, including the evolution of drug resistance in bacteria.

Shee et al. have developed a new method for detecting double-strand breaks in both bacterial and mammalian cells. The method involved combining a natural virus protein called Gam with a fluorescent protein called GFP (short for green fluorescent protein) to make a fusion protein called GamGFP. Gam was chosen because it binds only to double-strand breaks, traps double-strand breaks, and does not bind to any proteins. Genetic engineering techniques were used to introduce GamGFP into cells, with DNA breaks in these cells showing up as fluorescent spots when viewed under a microscope.

Shee et al. used this approach to detect double-strand breaks in both *Escherichia coli* cells and mammalian cells, and to measure the rate of spontaneous DNA breakage in *E. coli*. The number of double-strand breaks in *E. coli* was proportional to the number of times the cells had divided, which provides support for DNA replication-dependent models of spontaneous DNA breakage.

The GamGFP method also provided various insights into DNA breaks in mouse and human cells. In particular, Shee et al. found evidence for a mechanism of DNA breakage that appears to be specific to primates. This mechanism involves an enzyme that is only found in the innate immune system of primates removing an amine group from a cytosine. In future, this approach might allow the trapping, mapping and quantification of DNA breaks in all kinds of cells, and the highly specific way GamGFP binds to breaks could make it the preferred tool for studying DNA breakage in mammalian cells.

**DOI:** http://dx.doi.org/10.7554/eLife.01222.002

## Introduction

DNA double-strand breaks (DSBs) are the most genome-destabilizing DNA damage (Jackson and Bartek, 2009). ‘DSBs’ is used here as a collective term that includes two-ended structures (DSBs, e.g., as caused by double-strand endonucleases or ionizing radiation) and single double-stranded ends of DNA (DSEs, or one-ended DSBs), such as are caused by replication-fork collapses (Kuzminov, 2001). We use ‘DSE’ to refer to each single DSE in a two-ended DSB and to the sole DSE in a one-ended DSB. DSBs (one- and two-ended) promote deletions, genome rearrangements (Hastings et al., 2009), chromosome loss (Paques and Haber, 1999), and point mutations (Harris et al., 1994; Rosenberg et al., 1994; Strathern et al., 1995). DSB-induced genomic instability promotes cancer (Negrini et al., 2010) and genetic diseases (O’Driscoll and Jeggo, 2006), evolution of antibiotic resistance (Cirz et al., 2005) and of pathogenic bacteria (Prieto et al., 2006) including in biofilms (Boles and Singh, 2008). The latter reflect the role of DSBs in inducing mutagenesis and genome rearrangement under stress, which may accelerate evolution of bacteria (Al Mamun et al., 2012; Rosenberg et al., 2012), and human cancer cells (Bindra et al., 2007). DSBs are implicated in mutation hotspots in cancer genomes (Nik-Zainal et al., 2012; Roberts et al., 2012). Breaks induced by ionizing radiation and alkylating drugs are used as anti-cancer therapy, and conversely DSBs are likely to foretell genomic instability that drives malignancy (Negrini et al., 2010). Despite the importance of DSBs to many biological processes, quantification of DSBs has been limited. Moreover, although some mechanisms of DSB formation are being explicated (Merrikh et al., 2012), the main mechanisms underlying spontaneous DNA breakage in bacterial (Pennington and Rosenberg, 2007) and human cells (Vilenchik and Knudson, 2003; Kongruttanachok et al., 2010) remain elusive.

DSBs have been quantified via neutral sucrose gradients (e.g., Bonura and Smith, 1977), or pulse-field gels (PFGE) (Michel et al., 1997), neither of which routinely detects DSBs present in fewer than ∼10% of a population of molecules, far above DSB levels that occur in cells spontaneously (Pennington and Rosenberg, 2007). The standard single-cell gel electrophoresis (‘comet’) assay (Olive et al., 1990) detects single-strand (ss) DNA nicks and DSBs, and thus is not specific to DSBs, whereas the neutral comet assay (Wojewodzka et al., 2002) is DSB-specific, but lacks sensitivity. The terminal transferase dUTP nick end-labeling (TUNEL) assay detects free ends of DNA, and so (nonspecifically) labels both ssDNA nicks and DSBs (Gavrieli et al., 1992).

Cytological assays for foci of DSB-repair proteins identify locations of DSBs in situ via surrogate markers γ-H2AX (Rogakou et al., 1999), Mre11, Rad50 (Maser et al., 1997), Rad51 (Haaf et al., 1995), Rad52 (Liu et al., 1999), BRAC1 (Scully et al., 1997), Ku80/70 (Koike et al., 2011), and 53BP1 (Rappold et al., 2001) in eukaryotes, and RecA (Renzette et al., 2005), RecFON (Kidane et al., 2004), and bacterial Ku (Kobayashi et al., 2008) in bacteria. Only some of these may be DSB-specific. γ-H2AX and 53BP1, the most commonly used DSB markers in mammalian cells, are indirect markers. Antibodies to γ-H2AX and 53BP1 detect a modified histone and a DNA repair protein respectively, rather than DNA ends, and are likely to label sites not currently possessing a DSB. γ-H2AX is a histone variant phosphorylated at sites of DNA damage during DNA damage signaling. γ-H2AX spreads over up to ∼2-Mbp regions that comprise 500 to 8000 γ-H2AX molecules, so does not pinpoint DSB sites (Rogakou et al., 1999). Numbers of γ-H2AX foci induced by DNA damage may not represent true numbers of DSBs (Bouquet et al., 2006), and γ-H2AX focus formation is variable and can occur at non-DSB sites (Han et al., 2006). Thus, γ-H2AX may not always signify a physical break. 53BP1 is a non-homologous end-joining (NHEJ) protein and forms nuclear foci upon ionizing radiation (IR) treatment, dependently on several histone modifications including γ-H2AX, H2A/X ubiquitylation and H4K20 methylation (Lukas et al., 2011b; Fradet-Turcotte et al., 2013). Because these histone modifications do not exist equally throughout the genome, the efficiency and DSB-specificity of 53BP1 are not known. Moreover, γ-H2AX, and all histological markers provide ‘snapshots’ of fixed cells and do not allow the possibility of measuring accrual of DSBs over time. Although Ku is likely to be most specific for DSBs via its function in NHEJ (Taccioli et al., 1994), Ku also functions at telomeres (Gravel et al., 1998) and appears to interact with RNA polymerase II (Dynan and Yoo, 1998), raising questions about its specificity. Ku also binds ssDNA nicks, gaps, and regions of transition between single- and double-stranded structures, supporting concerns about its specificity (Paillard and Strauss, 1991; Blier et al., 1993).

In bacteria, RecA binds ssDNA assisted by RecF. Neither is specific for DSEs of DNA. Bacterial Ku has also been used to visualize DNA damage (Kobayashi et al., 2008). Quantification of DSBs in living *E. coli* was achieved by measuring the RecB-dependent (DSB-specific) induction of the SOS response in individual cells by flow cytometry (Pennington and Rosenberg, 2007). Although this assay allowed quantification of rates of formation of living cells with DSBs (Pennington and Rosenberg, 2007), it could not determine the numbers of DSBs per cell. Thus, to date, neither precise rates of spontaneous DNA breakage in living cells nor the mechanisms that underlie most spontaneous DNA breakage are known, even in *E. coli*.

DSBs can arise by several different mechanisms, many involving DNA replication. Replication-fork collapse at ssDNA nicks creates one-ended DSBs (Kuzminov, 2001) (illustrated below). Collisions of the replisome with transcription complexes (Merrikh et al., 2012) and other proteins (Gupta et al., 2013) cause DNA breakage. In stationary-phase (non-replicating) cells, RNA/DNA hybrids left by transcription (R-loops) promote DNA breakage apparently by priming replication forks that then collapse at ssDNA nicks (Wimberly et al., 2013). Though replication can generate DSBs, whether it is the principle generator of DSBs spontaneously, in cells/sites not specifically engineered to maximize collisions, has not been addressed. Similarly, spontaneous 53BP1 foci can be detected in G1 (non-replicating) human cells (Lukas et al., 2011a). Given the uncertainly of the DSB-specificity of 53BP1, whether these reflect genuine DSBs formed outside of S phase, when most replication occurs, is unclear.

Additional processes may generate DSBs in human cells. Most humans express up to nine primate-specific ssDNA deaminases, AID, APOBEC1, and seven distinct APOBEC3s, all of which convert DNA cytosines to uracils (Conticello et al., 2007). In human, uracils in DNA can be processed by UNG2 into abasic sites and subsequently into ssDNA nicks by APEX. Such nicks could potentially produce DSBs via opposing strand nicks or replication-fork collapses, as occurs with uracil excision from DNA in *E. coli* (Kuzminova and Kuzminov, 2008). Although DSBs have been inferred and associated with AID for Ig translocations in some B-cell cancers (Robbiani and Nussenzweig, 2013), DSBs have yet to be identified directly. DSBs have also been inferred as intermediates in the generation of strand-biased cytosine mutation clusters in many cancers (Nik-Zainal et al., 2012; Roberts et al., 2012; Burns et al., 2013b). This inference is further supported by the demonstration of similar-sized mutation hotspots targeted to DSBs created by I-SceI endonucleolytic cleavage in *E. coli* (Shee et al., 2012), and by similar mutation clustering in yeast cells exposed to alkylating agents or engineered to express various human DNA deaminases (Roberts et al., 2012; Taylor et al., 2013).

In this study, we develop engineered proteins for specific detection of DSBs in bacterial and mammalian cells, and use them to illuminate spontaneous DNA breakage in both. We created fluorescent-protein fusions of the highly DSE-specific (Williams and Radding, 1981; Akroyd and Symonds, 1986; Abraham and Symonds, 1990) Gam protein of phage Mu for detection of DSBs as foci upon its expression from the *E. coli* chromosome, or from vectors in mammalian cells. Gam is the ortholog of eukaryotic and bacterial Ku (d’Adda di Fagagna et al., 2003), but, unlike Ku proteins, does not perform DNA repair reactions nor bind any other known protein. During phage infection, Gam binds and protects ends of linear phage DNA, preventing degradation by host exonucleases (Akroyd and Symonds, 1986). Biochemically, Mu Gam is a highly specific DSE-binding protein (Williams and Radding, 1981; Abraham and Symonds, 1990). We demonstrate the utility of regulatable GamGFP fusion proteins for detecting DSBs in *E. coli* and in mammalian cells and use them to determine the rates of spontaneous DNA breakage in *E. coli*. Moreover, we track the origins of spontaneous DSBs in live, proliferating *E. coli*. We find precise correlation of DSBs with the numbers of divisions, implying that replication-dependent mechanism(s) underlie most spontaneous DNA breakage. In human and mouse cells, we show that GamGFP labels DSBs and we provide evidence that—(i) GamGFP competes with Ku for DSBs; (ii) 53BP1 appears less specific for DSBs than GamGFP; (iii) GamGFP inhibits end resection at DSBs; (iv) DNA cytosine deamination produces DSBs in human cells, identifying a potentially primate-specific mechanism of DNA breakage; and (iv) G1 cells show multiple clustered foci, implying that some spontaneous DNA breakage occurs outside of S phase when most replication takes place.

## Results

### Production of functional GamGFP from the *E. coli* chromosome

**Figure 1.**
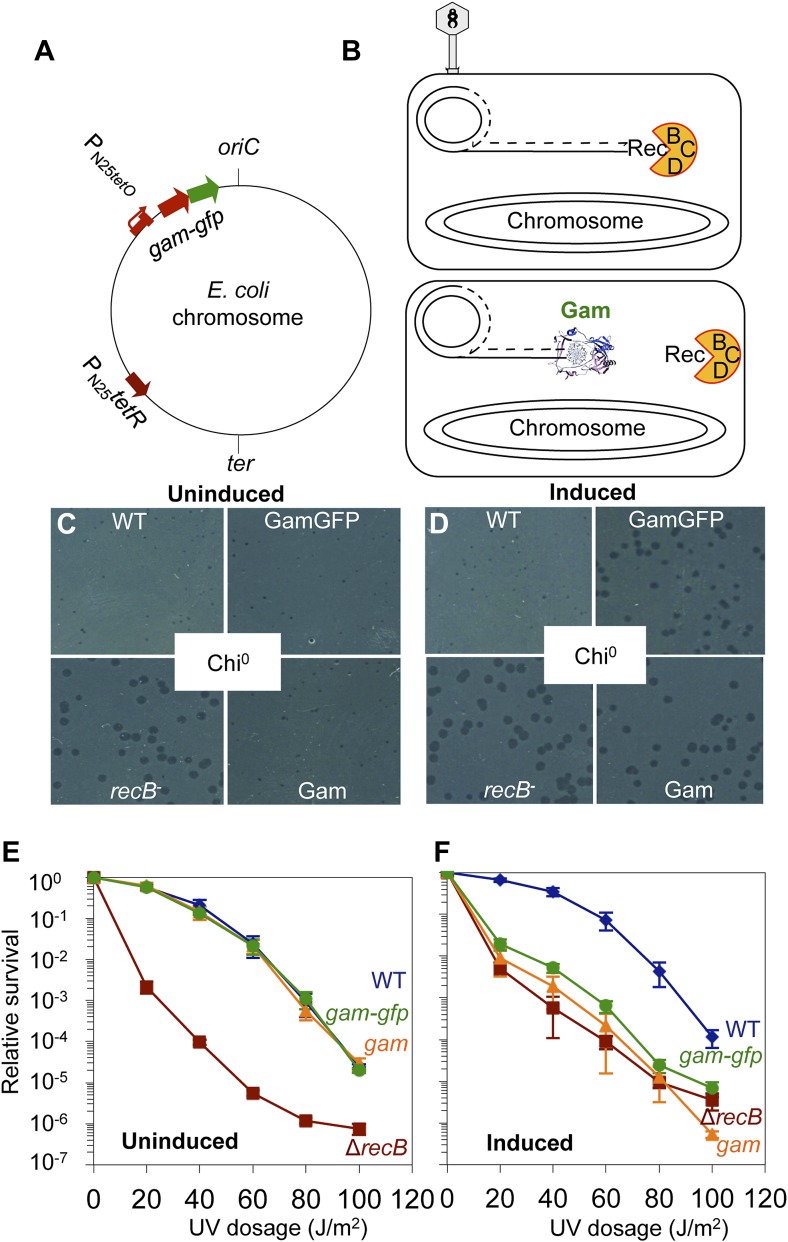
GamGFP production mimics *recB* double-strand-exonuclease defect. (**A**) Doxycycline-inducible *gam-gfp* fusion construct in the *E. coli* chromosome. Constitutively produced TetR protein represses the P_N25*tetO*_ promoter, which produces GamGFP upon doxycycline induction. *oriC*, origin of replication; *ter*, replication terminus; arrows, directions of transcription. (**B**) Phage λ assay for end-blocking activity by Mu Gam and GamGFP. Rolling-circle replication of phage λ*red gam* is inhibited by *E. coli* RecBCD, which causes small plaques of λ*red gam* on wild-type *E. coli* (Smith, 1983). Mu Gam protein binds and protects DNA ends from RecBCD exonuclease activity (Akroyd and Symonds, 1986) and so is expected to allow rolling-circle replication of λ*red gam* and therefore allow formation of large plaques. (**C**) λ*red gam* plaques are small on *recB*^*+*^ (WT) and large on *recB*-deficient cells (*recB*^*-*^). Plaques produced on WT cells carrying *gam* and *gam-gfp* are small when Gam and GamGFP proteins are not produced (Uninduced). (**D**) λ*red gam* produce large plaques on WT cells if Gam or GamGFP are produced (Induced). (**E**) UV sensitivity of *E. coli recB*-null mutant compared with *recB*^*+*^(WT), and uninduced *gam* and *gam-gfp* carrying cells. WT (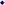), *recB*^−^ (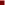), WT GamGFP, (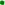); WT Gam, (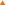). (**F**) Induction of Gam or GamGFP with 200 ng/ml doxycycline causes UV sensitivity similar to that of *recB-*null mutant cells, indicating that Gam or GamGFP block RecBCD action on double-stranded DNA ends. WT, SMR14327; *recB*, SMR8350; WT GamGFP, SMR14334; WT Gam, SMR14333. Representative experiment performed three times with comparable results. **DOI:** http://dx.doi.org/10.7554/eLife.01222.003

We constructed a regulatable chromosomal expression cassette of Mu *gam* and a Mu *gam-gfp* fusion gene in the *E. coli* chromosome, controlled by the doxycyline/tetracycline-inducible P_N25*tetO*_ promoter (‘Materials and methods’, Figure 1A). Promoter-only and GFP-only controls were also constructed. Production of GFP, Gam, and GamGFP were verified by SDS-PAGE and western analyses (Figure 1—figure supplement 1). *gam-gfp* and a derivative *gam-EmGFP* fusion gene were sub-cloned into a mammalian expression system using the *E. coli* chromosomal construct as a template.

**Figure 1—figure supplement 1.**
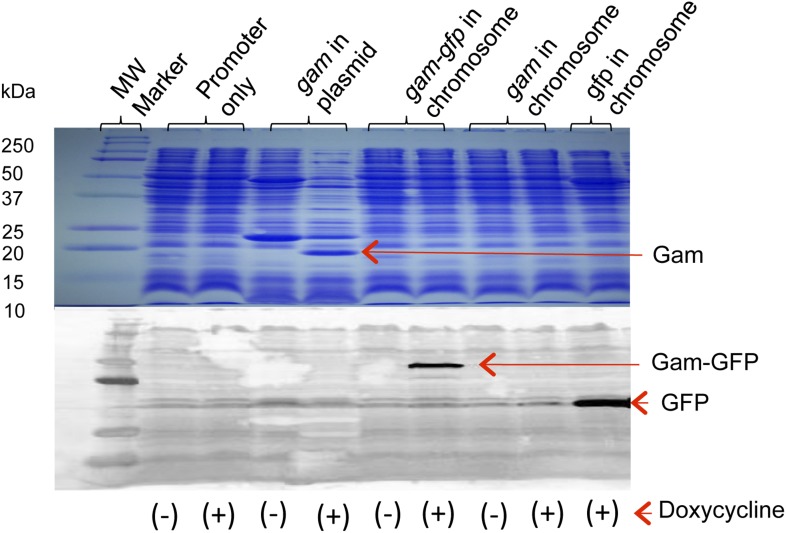
Production of Gam and GamGFP fusion proteins in *E. coli*. Doxycycline induction and detection of plasmid-borne Gam and chromosomally encoded GamGFP and GFP are performed by Coomassie blue staining following electrophoresis (Gam) or by western blot immunodetection (GamGFP, GFP), in upper and lower panels, respectively. Cultures grown, as described in ‘Materials and methods’, were incubated in the presence or absence (+ or −) of 100 ng/ml doxycycline. For the western blot, protein was visualized using antibodies against GFP. Arrows indicate positions of Gam*,* GFP, and GamGFP within the gel. Molecular weights of protein standards are indicated to left. Strains are: promoter only, SMR14311; *gam* in plasmid, SMR13908; chromosomal *gam-gfp*, SMR14334; chromosomal *gam*, SMR14333; and chromosomal *gfp*, SMR14332. **DOI:** http://dx.doi.org/10.7554/eLife.01222.004

**Figure 1—figure supplement 2.**
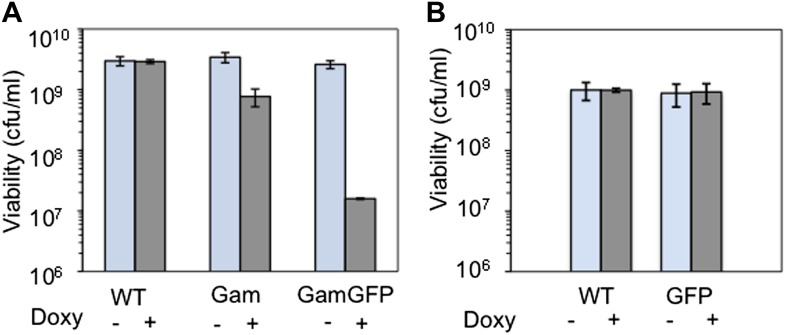
Long-term GamGFP production reduces *E. coli* viability. Greater viability loss with GamGFP than Gam implies that GamGFP is a superior DSE trap. (**A**) We quantified the effect of long-term Gam and GamGFP production on cell viability by inducing Gam or GamGFP production briefly in split log-phase cultures, then plating them for viable colony-forming units (cfu) on inducing or non-inducing solid medium with long-term overnight incubation. Saturated LBH cultures of SMR14327 (WT), SMR14333 (Gam) and SMR14334 (GamGFP) were diluted 1:100 in fresh LBH medium and grown shaking at 37°C for 90 min, then either induced with 200 ng/ml of doxycycline or not, and grown for an additional 2 hours shaking at 37°C prior to plating for cfu on LBH solid medium with or without 200 ng/ml doxycycline. The colonies were scored after overnight incubation at 37°C. We observe that induced cultures of the Gam- and GamGFP-producing strains show, respectively, 32 ± 9% and 0.47 ± 0.06% the number of viable cfu as either WT cells with no *gam* gene or uninduced *gam-* or *gam-gfp-*containing cells (mean ± SD, three experiments). These data imply that DSBs bound by GamGFP are not repaired or are repaired inefficiently. Whereas the viability of Gam producers is similar to that of liquid cultures of *E. coli recBC* DSB-repair*-*defective cells, which typically contain ∼30% viable cells (e.g., Miranda and Kuzminov, 2003), GamGFP producers have lower viability. These data suggest that GamGFP is a more permanent DSE-trap and blocker of repair than Gam is, and that there is residual RecBC-independent DSB repair in *recBC*-defective cells. We speculate that the GFP moiety might confer more permanence to the GamGFP binding of DSBs either because the GamGFP protein inherently possesses a reduced dissociation constant or perhaps because the GFP moiety instigates multimerization with other GFP moieties in other GamGFP molecules present in the cell. This could both confer its outstanding focus-forming ability and might additionally retard end dissociation. (**B**) We find no cfu-reducing effect of production of GFP alone. **DOI:** http://dx.doi.org/10.7554/eLife.01222.005

We show that chromosomally encoded Gam and GamGFP are functional in *E. coli* by demonstrating that their production blocks the action of RecBCD, a highly DSE-specific dsDNA exonuclease, in two assays.

First, phage lambda (λ) lacking its own Gam protein (which, unlike Mu Gam, is a RecBCD-binding protein) and Red recombination proteins forms small plaques because RecBCD DSE-dependent exonuclease prevents λ rolling-circle replication (Smith, 1983) (Figure 1C). By contrast, λ*red*^*−*^ *gam*^*−*^ forms large plaques on *recB-* (or *recC* or *recD*)-defective *E. coli* because rolling-circle replication occurs (Smith, 1983) (Figure 1C,D). We show that wild-type *E. coli* producing either Mu Gam or GamGFP allow large plaque formation by λ*red*^*−*^ *gam*^*−*^, equivalent to those seen on *recB-*null-mutant *E. coli* (Figure 1D). We conclude that chromosomally encoded Mu Gam and GamGFP block RecBCD exonuclease activity, implying that they are functional for the Mu Gam DSE-binding activity (Williams and Radding, 1981; Akroyd and Symonds, 1986; Abraham and Symonds, 1990).

Second, *recB*-null cells are highly sensitive to ultraviolet (UV) light (Willetts and Clark, 1969) because they are DSB-repair deficient, and UV-induced damage can lead to DSBs (Bonura and Smith, 1975), which are lethal if not repaired (Figure 1E). We find that induction of Gam or GamGFP in wild-type *E. coli* creates a phenocopy of the *recB* UV sensitivity that is almost identical to that of *recB*^−^ cells (Figure 1F). These data, and those from the λ assay, show that Gam or GamGFP blocks RecBCD DSE-dependent exonuclease/DSB-repair activity, implying that chromosomally produced GamGFP binds DSEs of DNA in living *E. coli*.

Additionally, we also show that long-term Gam or GamGFP production confers poor viability, expected for DSB-repair-deficient cells, supporting a complete block to DSB repair (Figure 1—figure supplement 2). *recBC* null-mutant cells show severe loss of viability, with only about 30% of cells present in liquid cultures being viable and able to produce colonies (e.g., Miranda and Kuzminov, 2003). This results, presumably, from reduced repair of spontaneous DSBs. In Figure 1—figure supplement 2, we show that long-term Gam production causes a similar low viability of 32 ± 9% viable cells relative to uninduced or wild type (WT) cells. GamGFP shows even further reduced viability (0.5% ± 0.06%), even though GFP production alone causes no reduction in viability. As discussed (Figure 1—figure supplement 2A legend), the data imply that GamGFP is a better blocker of DSB repair than Gam, and that it blocks RecBC-dependent and also one or more RecBC-independent, residual DSB-repair pathways.

### GamGFP forms foci at two-ended DSBs

**Figure 2.**
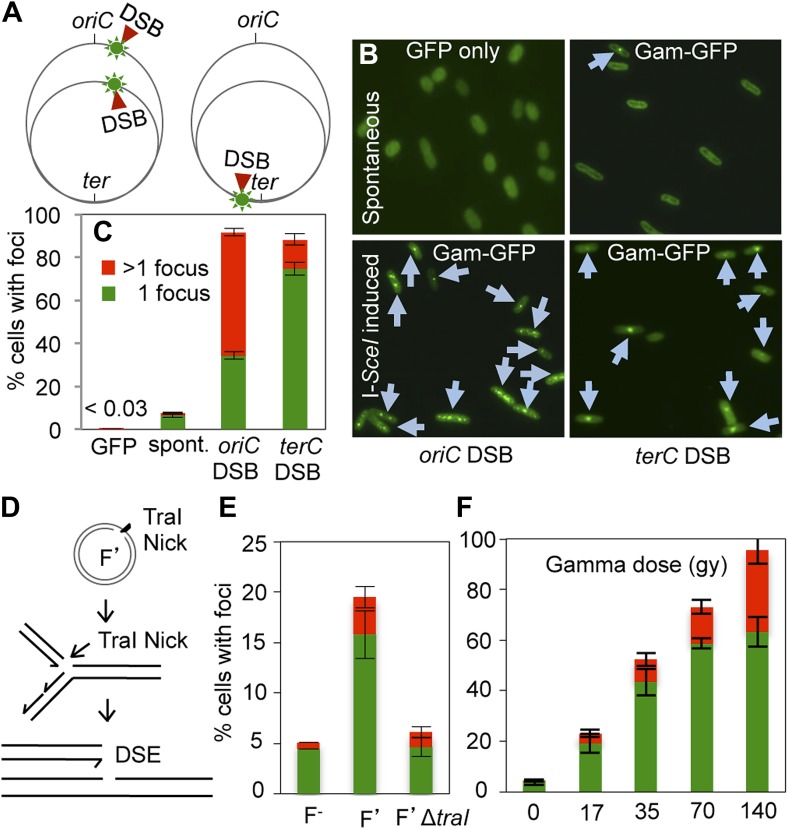
GamGFP foci at DSBs in living *E. coli*. (**A**) Strategy. In log-phase replicating *E. coli*, cells have more copies of origin (*oriC*)-proximal than terminus (*ter*)-proximal DNA and so will have more DSBs per cell when cleaved by chromosomally encoded I-*Sce*I (Ponder et al., 2005) at a cutsite (red arrow/green flash) near *ori* than near *ter*. (**B**) Representative data (arrows indicate foci). (**C**) Quantification from multiple experiments shows correlation of GamGFP foci with numbers of DSBs per cell. Cells have >1 focus when cleaved by I-*Sce*I near *ori*, usually 1 focus per cell when cleaved by I-*Sce*I near *ter*, far fewer cells with foci when only spontaneous DSBs are present (no I-*Sce*I cleavage), and <0.03% of cells with foci when GFP alone is produced. *E. coli* strains: GFP only, SMR14332; GamGFP, SMR14350; *oriC* DSB, SMR14354; *ter* DSB, SMR14362. Error bars, ± SEM. (**D**) Strategy: a site-specific ssDNA nick made by TraI ssDNA endonuclease at *oriT* in the F plasmid becomes a one-ended DSB upon replication by fork collapse (Kuzminov, 2001). (**E**) TraI-dependent GamGFP foci imply that GamGFP detects one-ended DSBs. Cells with no F plasmid (F^−^), an F′ plasmid encoding TraI (F′), or an isogenic *traI*-deleted F′ (F′Δ*traI*): strains SMR14015, SMR16387, and SMR16475. (**F**) GamGFP foci are correlated with dose of DSB-producing γ-radiation. Figure 2—figure supplement 2A shows linear correlation of foci with dose. Strain, SMR14350. Cells with 1 focus, green; >1 focus, red. **DOI:** http://dx.doi.org/10.7554/eLife.01222.006

We used the chromosomal regulatable I-*Sce*I double-strand endonuclease (Gumbiner-Russo et al., 2001; Ponder et al., 2005) and chromosomal I-*Sce*I cutsites (I-sites) (Shee et al., 2012) to make site-specific DSBs in the *E. coli* chromosome and show that GamGFP forms foci at DSBs in living cells (Figure 2A–C). First, when GamGFP is produced for 3 hr in cells without I-*Sce*I endonuclease, spontaneous foci (presumably reflecting spontaneous DSBs, validated below) are visible in ∼7.5% of cells, a number higher than the 2.1% of cells with DSBs reported in a previous assay (Pennington and Rosenberg, 2007). This discrepancy, we show below, reflects different growth medium (which affects growth rate and the number of chromosomes per cell) from that of the previous assay. Second, GamGFP forms foci in almost all cells when I-*Sce*I is induced in cells carrying an I-*Sce*I cutsite (Figure 2B,C), and not in cells expressing only the enzyme (no cutsite, not shown), or carrying the cutsite but no enzyme (Figure 2C, “spontaneous”). These data imply that DSBs underlie foci. Third, we varied the number of DSBs per cell by using rapidly growing cells with the I-site either near the replication origin (*ori*) (more DNA copies, so more DSBs upon I*-Sce*I induction) or near the replication terminus (fewer DNA/I-site copies, so fewer DSBs, Figure 2A). qPCR showed ∼2–3 times more *ori-* than *ter-*proximal DNA copies under these conditions (Figure 2—figure supplement 1). We find that although the number of cells with foci is the same with the I-site near *ori* as *ter*, the number of foci per cell is significantly greater when cleavage is near *ori* than when it is near *ter* (Figure 2B,C). Whereas the cells with an *ori-*proximal I-site had 57 ± 2% of cells with >1 focus, those with the *ter*-proximal I-site had a significantly lower 14 ± 3% of cells with >1 focus (p*=*0.0001, Student’s *t* test). The data show that foci form proportionately to the number of DSBs per cell and imply that foci form at the DSB sites (supported independently below).

**Figure 2—Figure supplement 1.**
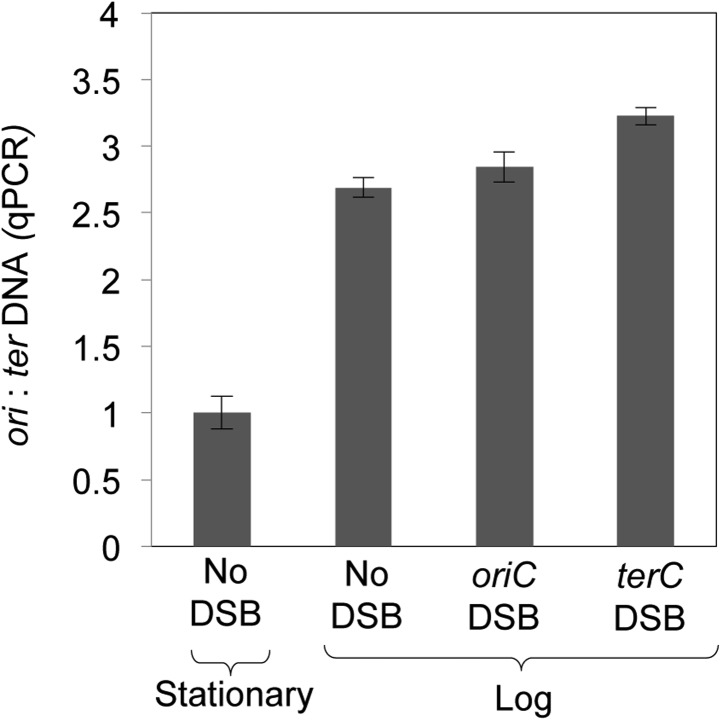
Quantitative real-time PCR shows ∼three-fold more DNA copies near *ori* than *ter* in log-phase, regardless of I-*Sce*I cleavage, implying that some cells have two and some have four *ori*:*ter* regions. **DOI:** http://dx.doi.org/10.7554/eLife.01222.007

**Figure 2—figure supplement 2.**
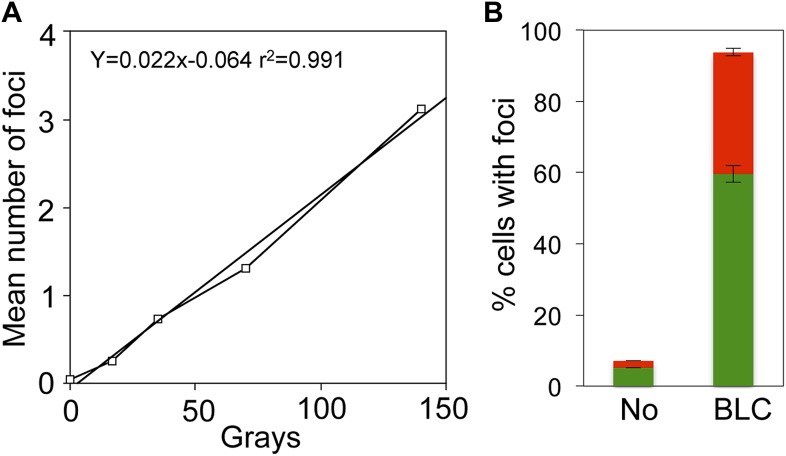
Linear gamma-ray dose-response and bleomycin induction of GamGFP foci in *E. coli*. (**A**) Numbers of GamGFP foci are linearly correlated with dose of DSB-producing γ-irradiation. Numbers of foci at different doses of γ-irradiation were calculated from the data displayed in Figure 2F. (**B**) GamGFP foci form in response to bleomycin induced DSBs in living *E. coli*. Twenty µg/ml bleomycin (BLC) promotes GamGFP foci. Green, 1 focus per cell; red, >1 focus per cell. **DOI:** http://dx.doi.org/10.7554/eLife.01222.008

These data also imply that a single focus occurs at each I-*Sce*I-induced DSB: one focus for each of the two DSEs present in the DSB. Because two foci per cell can be detected when more than one chromosome has a DSB (Figure 2A–C,E,F), we infer that there is not an inherent limit by which only one focus can form in a cell. Rather the two DSEs present in a single DSB appear to be kept close enough to each other, either by GamGFP or perhaps by an *E. coli* DNA-repair or other protein(s), that only a single focus is visible.

### GamGFP detects one-ended DSBs

DSBs formed by I-*Sce*I cleavage contain two double-stranded DNA ends (DSEs). Our data suggest that GamGFP binds at both DSEs next to each other and forms a single focus. By contrast a one-ended DSB is expected to result when replication encounters a ssDNA nick (Figure 2D) via fork collapse (Kuzminov, 2001), a postulated frequent occurrence. We mimicked such fork collapses using the constitutive ssDNA nicking that occurs in the *E. coli* F conjugative plasmid. F plasmids are constitutively nicked by TraI ssDNA endonuclease, at a specific site, *oriT,* to initiate conjugal DNA transfer (Traxler and Minkley, 1988)*.* ssDNA nicks become single DSEs (one-ended DSBs) upon replication-fork collapse (Kuzminov, 2001) (Figure 2D). We observe a TraI-dependent, four-fold increase in GamGFP foci in F′-carrying cells compared with isogenic F^−^ cells (Figure 2D,E), implying that GamGFP also labels one-ended DSBs, in addition to two-ended DSBs such as those created by double-strand endonuclease I-*Sce*I.

### DSB-detection efficiency

We used two known DSB-inducing treatments to quantify the efficiency of GamGFP focus formation relative to known efficiencies of DNA breakage by one of these agents, and to generalize our conclusions with the other. Gamma rays are widely used to induce DSBs, and the DSB load per gray (Gy) of ionizing radiation (IR) in *E. coli* is documented (Bonura and Smith, 1977). We find that focus formation is linearly related to gamma-ray dose over the range of 0–140 Gy (r^2^ = 0.991) (Figure 2F, Figure 2—figure supplement 2A). We observed 3.07 foci per cell given 140 Gy (3.12 foci/cell at 140 Gy less 0.043 foci/cell at 0 Gy), or 0.022 foci/cell/Gy. Comparing this with the figure obtained by sucrose sedimentation of 0.031 DSBs/cell/Gy for *E. coli* (Bonura and Smith, 1977)*,* we infer an efficiency of detection of DSBs as GamGFP foci of 71% (0.022/0.031 = 0.71).

The 71% efficiency of detection should be considered a rough estimate because although we used identical growth medium and conditions to those used previously (Bonura and Smith, 1977), and the number of DSBs per *E. coli* cell per Gy is expected to be constant, we did not perform independent measurement of DSBs after IR by neutral sucrose gradients as per Bonura and Smith (1977). Therefore, some variation is possible. However, using an independent method below, we obtained a roughly similar estimate of efficiency (see *GamGFP pinpoints subgenomic locations of DSBs in E. coli*).

The smaller number of foci per cell with the zero dose (0.043) than observed in Figure 2C (spontaneous, 0.075), reflects the poorer growth medium used in these IR experiments: M9 0.4% glucose exactly as per Bonura and Smith (1977) vs rich LBH medium in Figure 2C. *E. coli* produces more chromosome copies per cell in LBH than M9 medium (Pennington, 2006), and so is expected to have more spontaneous DSBs per cell in rich medium than poor (also shown below).

Bleomycin also induces DSBs (Hecht, 2000). We see that log-phase cells treated with bleomycin show significantly increased foci. About 60% have one focus and ∼34% have ≥2 foci (94% of cells with foci total), about 14-fold higher than spontaneous focus levels (Figure 2—figure supplement 2B). These results generalize the DSB-labeling activity of GamGFP, and the results with gamma rays indicate that a high proportion, ∼71%, of DSBs are detected by GamGFP in *E. coli*.

### GamGFP pinpoints subgenomic locations of DSBs in *E. coli*

**Figure 3.**
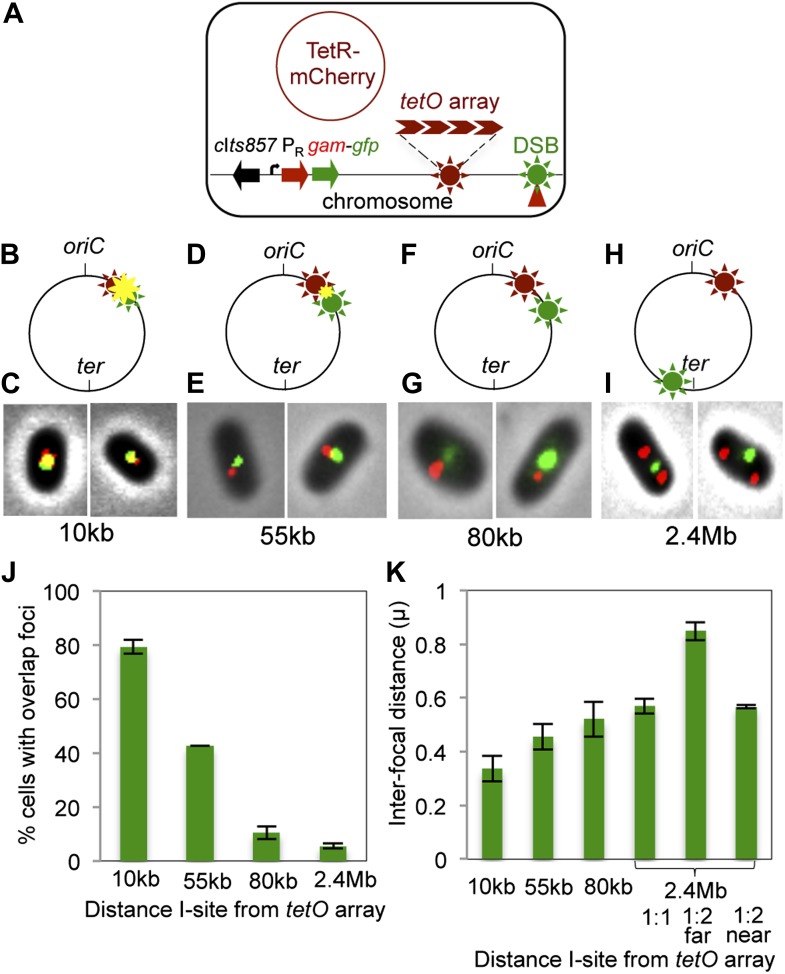
Subcellular/subgenomic localization of DSBs in living *E. coli*. (**A**) Strategy: we varied the location of I-*Sce*I cleavage sites (I-sites) in different strains relative to a fixed-position chromosomal TetR-mCherry-bound *tetO* array, with GamGFP temperature inducibly produced from chromosomal λP_R_ (*c*I*ts857* P_R_*gam-gfp*). Red circle, plasmid that produces TetRmCherry. (**B**–**H**) Diagrams of *E. coli* chromosomes with inducible I-*Sce*I endonuclease and I-sites engineered (**B**) 10 kb, (**D**) 55 kb, (**F**) 80 kb, and (**H**) 2.4 Mb from the *tetO* array. Co-localization of TetR-mCherry (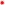) and GamGFP foci (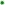) results in a yellow focus (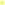). (**C**, **E**, **G**, **I**) Representative fluorescence microscopy results show co-localization of mCherry and GamGFP (yellow foci) at 10 kb (**C**), and non-overlapping foci at 55 kb (**E**), 80 kb (**G**), 2.4 Mb (**I**) in strains SMR16600, SMR16711, SMR16713, and SMR16606. (**J**) Percentage of cells with yellow overlapped foci at each distance. (**K**) Mean interfocal distances. At 2.4 Mb, there were frequently two red foci per one green focus, reflecting more copies of *ori*- than *ter*-proximal DNA during replication. The greater interfocal distance (far) is plotted separately from the shorter (near), and cells with 1:1 ratios were counted separately. Data represent three independent experiments, error bars indicate SEM, with the number of cells counted in all three totaling: 298, 10 kb; 204, 55 kb; 333, 80 kb; and 1347, 2.4 Mb. **DOI:** http://dx.doi.org/10.7554/eLife.01222.009

We show that GamGFP foci indicate the subcellular/subgenomic locations of DSBs in *E. coli* using site-specific I-*Sce*I cleavage combined with a fixed chromosomal tetracycline operator (*tetO*) array bound by a Tet repressor (TetR)-mCherry fusion protein, which forms a focus at a site near *oriC* (Figure 3A). In cells carrying this chromosomal-site label, we introduced an I-*Sce*I cutsite (I-site) either 10 kb, 55 kb, 80 kb or 2.4 Mb away in different strains (Figure 3B,D,F,H) and quantified co-localization of GamGFP and TetR-mCherry (representative data, Figure 3C,E,G,I). We find that GamGFP foci co-localize with the TetR-mCherry focus, producing a yellow focus, about 80% of the time with the I-site 10 kb from the array (Figure 3C,J). The remaining 20% of cells had average interfocal distances of ∼0.3 μm (Figure 3K). With 55 kb, 80 kb, and 2 Mb separating the I-site and *tetO* array, the mean distances between green and red foci increased to 0.45 μm, 0.52 μm, and to 0.57 μm respectively (Figure 3K) and the number of cells with overlapping (yellow) foci decreased (Figure 3J). With the most distant I-site, most cells had one green (*ter*-proximal) focus and two red (*ori*-proximal) foci with the average distance ∼0.84 μm (2.4 Mb far, Figure 3K) and 0.57 μm (2.4 Mb near, Figure 3K) between the single green focus and each of the two red foci. The percentages of co-localization were significantly different for 10, 55 and 80 kb (p=0.00003, 0.00001, and 0.00006, Student’s *t* test) and not between 80 kb and 2.4 Mb (p=0.053). These data imply that sites farther than 80 kb apart are not necessarily further apart in space within the bacterial nucleoid (3-D chromosome structure), at least not after the nucleoid has suffered double-strand cleavage. Interfocal distances were more variable and less significantly different than the proportion of co-localization, probably reflecting the dynamic nature of the chromosome within the nucleoids of these living cells. Our data show that co-localization of GamGFP and TetR-mCherry foci occurs when the cutsite is near to and not when it is far from the array. These results indicate first, that GamGFP can diagnose subgenomic locations of DSBs. Second, the data imply that beyond 80 kb, genomic locations are roughly the same physical distance apart in cleaved bacterial nucleoids (shown previously for sites >200 kb apart in uncleaved nucleoids [Wang and Sherratt, 2010]). Third, the data support the conclusion (above) that GamGFP foci occur at DSB sites.

These data also allow an independent (though equivocal) estimation of the efficiency of GamGFP focus formation at DSBs. Because red *tetO-*array foci label chromosomes, we can use the fraction of green (GamGFP) per red (chromosome) focus to approximate the efficiency of GamGFP focus formation at DSBs per chromosome. This method has the caveat that we do not know the efficiency of I-*Sce*I cleavage of the chromosomes under our induction conditions. Nevertheless, using the construct in which the *tetO* array and I-site are nearby, at 55 kb away, we observe 0.82 ± 0.03 (mean ± SD, three experiments) green per red focus, indicating a rough efficiency of 82% of DSBs with a focus. This is similar to our estimate of ∼71% of DSBs with a focus, above.

### Generation-dependence and rate of spontaneous DNA breakage in *E. coli*

Previously, we estimated the steady-state frequency of proliferating *E. coli* with one or more spontaneous DSBs to be between 0.5% and ∼2.1%, and from this derived rates of formation of break-carrying cells between 0.25% to ∼1% per generation (Pennington and Rosenberg, 2007). However, two problems cloud interpretation of the previous data. First, the previous method could not distinguish whether most spontaneous DSBs occur singly in cells or via multi-break catastrophes. Second, whether most spontaneous DSBs occur replication- and thus generation-dependently was unknown (reviewed in ‘Introduction’). GamGFP allowed solution of both problems.

**Figure 4.**
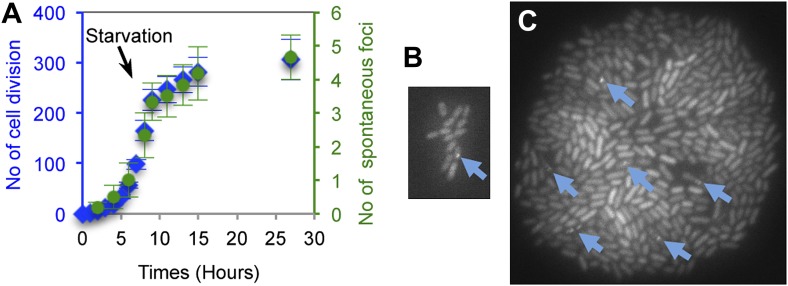
Generation-dependence of spontaneous GamGFP focus formation in proliferating *E. coli*. Log-phase GamGFP-pre-induced cells were loaded into a microfluidic chamber in which single cells anchor then divide to form single-cell-layer microcolonies. The numbers of cell divisions and appearance of spontaneous foci were captured with time-lapse photography. Rapid growth in glucose during the first 9 hr was followed by washing cells in the same medium lacking glucose for 18 hr to slow and halt cell divisions. (**A**) Spontaneous DSB foci are correlated with numbers of cell divisions. Summary of data for six cells that became microcolonies. Blue (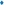), number of cell divisions; green (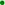), cumulative number of spontaneous foci that appear in each microfluidics micro-colony (mean ± SEM, six microcolonies). (**B**) Representative 2-hr micro-colony with a GamGFP focus (arrow). (**C**) Representative 15-hr micro-colony with GamGFP foci (arrows). **DOI:** http://dx.doi.org/10.7554/eLife.01222.010

First, time-lapse microfluidic imaging shows that most spontaneous DSBs form with precise correlation to numbers of cell divisions, and as above (e.g., Figure 2A–C) they form mostly 1 DSB per cell, not in multi-break catastrophes. In microfluidic chambers, we captured images of growing microcolonies from the 1-cell to ∼100-cell stage measuring divisions and appearance of spontaneous GamGFP foci while varying cell-division rates by withdrawal of glucose from the flowing medium (Figure 4, Figure 4—figure supplement 1). If spontaneous DSBs form independently of replication/generations, then the focus appearance might correlate with time not generations, whereas replication-dependent mechanisms of DSB formation predict correspondence with generations. In Figure 4A, cells that were kept dividing in log-phase for 9 hr, then shifted to no-glucose for an additional 18 hr, show severely slowed divisions after the shift, and a highly precise correspondence of the numbers of spontaneous DSB foci with numbers of cell divisions at all division rates (Figure 4A, Figure 4—figure supplement 1). To verify that the cells experiencing slow/no growth were still capable of forming GamGFP foci had breaks been present, we gave 20 μg/ml of DSB-producing agent phleomycin after 27 hr and found that 45 ± 5% (mean ± SEM) of cells then formed GamGFP foci (Figure 4—figure supplement 1, 32 hr). Thus, new DSBs could have been detected if they had formed. These results provide the first demonstration that most spontaneous DSBs in *E. coli* form generation-dependently and support replicative models for the origins of most spontaneous DSBs.

**Figure 4—Figure supplement 1.**
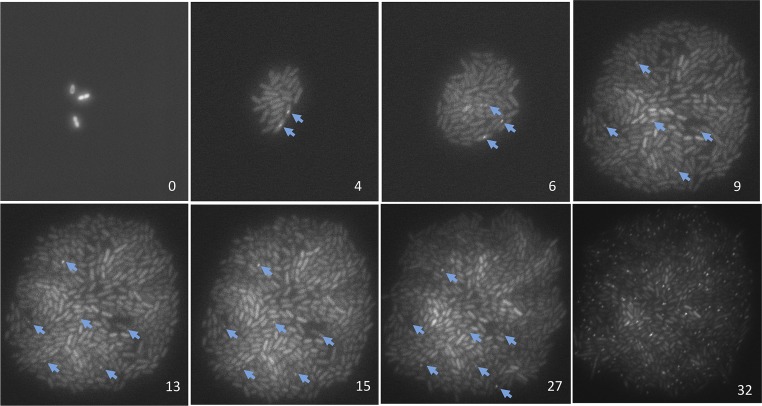
Representative data on the origins of spontaneous DSBs over time during growth, or growth retardation, visualized and quantified per ‘Materials and methods’, Microfluidics and time-lapse fluorescence microscopy of *E. coli*. Numbers in the lower right of each frame are hours after loading into the microfluidic chamber. GamGFP foci are indicated with arrows. Note that cells expressing *gam-gfp* show variation in the amount of GFP per cell documented for *E. coli* (Elowitz et al., 2002) and other cells expressing any fluorescent-protein gene. This variation represents stochastic variation in transcription and mRNA accumulation, but the data scored are foci (arrows). Cells were bathed in medium with glucose for 9 hr to allow log-phase growth, then cell divisions slowed and ultimately halted (Figure 4A) by switching to the same medium without glucose. Then at 27 hr, 20 µg/ml phleomycin was added to induce DSBs, visible as foci in 45± 5% of cells at 32 hr, to verify that had DSBs formed in the starving cells, they would have been visible as foci. These images were taken under very low-dose (30 ms) exposure to fluorescent light to minimize fluorescence-induced DNA damage (Ge et al., 2013) and possible induction of GamGFP foci. Control experiments summarized in ‘Materials and methods’ show that these brief pulses did not induce GamGFP foci (Microfluidics and time-lapse microscopy of *E. coli*, ‘Evidence that fluorescence exposure did not contribute to the spontaneous GamGFP foci scored)’. **DOI:** http://dx.doi.org/10.7554/eLife.01222.011

Second, the microfluidic data provide the rate of DNA breakage per cell division directly, as follows. Figure 4A shows a constant frequency of cells with a single focus (mean 0.0145 ± 0.006 SEM per cell division; none had >1 focus) independently of cell-division rate. In all six experiments summarized in Figure 4A, one focus appeared per cell, and cells with a focus did not divide further (probably because GamGFP is a DSE ‘trap’ that prevents repair of the break (Figure 1F) and shown for native Mu Gam protein in phage λ repair assays, Thaler et al. [1987]). Therefore, 0.0145 ± 0.006 foci per cell division represents the rate of formation of foci per division. Correcting this rate for ∼71% efficiency of detection of DSBs as foci (above), provides a rate of 0.021 ± 0.008 DSBs per cell division.

Third, the data obtained here additionally allow accurate interpretation of previous data from experiments that used flow-cytometric detection of cells with one or more DSBs (Pennington and Rosenberg, 2007) for a separate rate measurement as follows: (i) we now know that most of these cells have one DSB, not multi-break catastrophes and so can estimate real DSB formation rates; (ii) whereas previously, we assumed generation-dependence, a point that was not known, here we showed that spontaneous breaks do form generation-dependently (Figure 4). Thus we can translate the previously estimated rate to real rates of DSBs per cell division. Previously, the rate of formation of cells with ≥1 DSB per generation was estimated to be 0.01 (Pennington and Rosenberg, 2007). We can correct this for the number of DSBs per cell by noting that under the same growth conditions (exponential growth in minimal glucose) we observed 108 foci in 98 cells with foci, or ∼1.1 foci per focus-carrying cell. Applying this function to an estimate of 0.01 of cells producing ≥1 DSBs per generation (Pennington and Rosenberg, 2007), we have 0.01 × 108/98 = 0.011 DSBs per cell division. This is similar to the 0.021 ± 0.008 DSBs per cell division obtained from the microfluidic data above, and both are far lower than initial estimates (Cox et al., 2000), discussed below.

These rates hold for log-phase cells grown in minimal 0.1% glucose medium, in which most cells possess two chromosomes (Pennington and Rosenberg, 2007), the growth medium and condition used by Pennington and Rosenberg (2007). When growing in rich LBH medium, as we did in Figure 2A–C, replication is faster, the number of chromosomes per cell is increased, and the frequency of cells with DSB(s) is higher (Pennington, 2006). Similarly, we observed the higher 0.075 foci per cell in rich medium (Figure 2C). In the moderately richer M9 0.4% glucose medium used in the zero-dose of the IR experiments (Figure 2F), we observed the moderately higher frequency of 0.043 foci per cell. These data support the correlation between growth rate/the number of chromosomes per cell and spontaneous focus/DSB frequency or rate.

### GamGFP binds laser- and IR-induced breaks in mammalian cells and is inhibited by Ku

**Figure 5.**
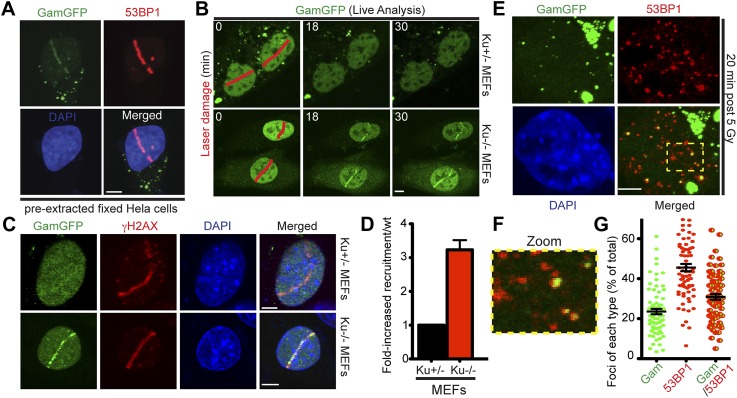
GamGFP marks DSBs in mammalian cells and is inhibited by Ku. (**A**) GamGFP co-localizes with 53BP1 on laser-induced DNA breaks. (**B**) Ku inhibits recruitment of GamGFP to laser-induced damage, live cells. (**C** and **D**) Ku inhibits recruitment of GamGFP, fixed cells. Mean ± SEM of three experiments, n >25 cells each. (**E**) GamGFP forms IR-induced foci in Ku80-defective MEFs. (**F**) Zoomed image from **E**. (**G**) IR-induced foci containing Gam only, 53BP1 only or both Gam and 53BP1 (>2600 total foci counted in three independent experiments). Error bars, SEM. Scale bars = 5 μm. **DOI:** http://dx.doi.org/10.7554/eLife.01222.012

We find that HeLa cells expressing GamGFP, in which DNA is sheared by a laser beam across the nucleus, display recruitment of fluorescence signal to the laser line (Figure 5A, Figure 5—figure supplement 1A). GamGFP co-localized with 53BP1 visualized by immunofluorescence staining in the same laser-treated and fixed samples (Figure 5A). We observed less robust GamGFP localization at laser damage in living (Figure 5—figure supplement 1A) than fixed (Figure 5A) HeLa cells, perhaps because pre-extraction of soluble GamGFP during fixation increases the apparent signal from the bound GamGFP (Figure 5—figure supplement 1B). We tested whether competition with Ku for DNA end-binding might be a factor affecting Gam localization to DSBs, as seen in yeast (d’Adda di Fagagna et al., 2003). We found ∼three-fold better labeling of laser-induced DSBs in Ku-deficient cells (lacking Ku80, also known as *Xrcc5*^*−/−*^; in which Ku70 is also downregulated, Taccioli et al., 1994) compared with heterozygous Ku80-competent cells (*Xrcc5*^*+/−*^) or *Lig4*^*−/−*^ (end-joining-defective but Ku-competent) mouse embryonic fibroblast cells (MEFs) (Figure 5B–D, Figure 5—figure supplement 2). Because GamGFP is inhibited by Ku even in end-joining-defective cells lacking LigIV (Figure 5—figure supplement 2), we infer that competition with Ku reduces GamGFP recruitment to DSBs independently of NHEJ, not that end-joining reduces the numbers of DSBs present for GamGFP to bind. These data indicate that GamGFP labels DSBs in mammalian cells.

**Figure 5—figure supplement 1.**
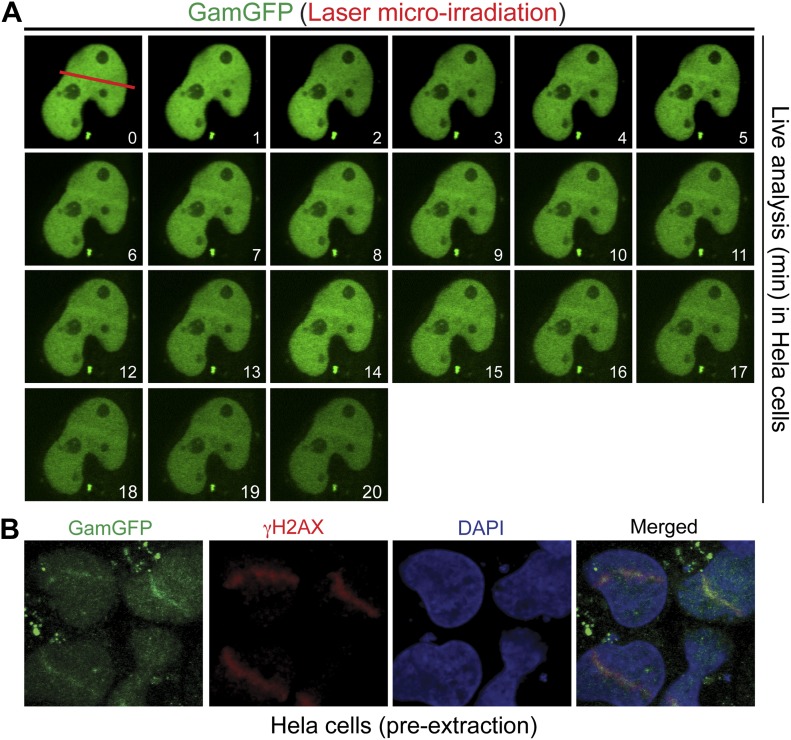
GamGFP marks DSBs in mammalian cells. (**A**) Live analysis of GamGFP localization to laser-induced DNA damage. Hela cells producing GamGFP were laser damaged along the cell track indicated by the red line at 0 min (m) and images were taken at the indicated times as shown. (**B**) GamGFP co-localizes with γH2AX in fixed Hela cells. **DOI:** http://dx.doi.org/10.7554/eLife.01222.013

**Figure 5—Figure supplement 2.**
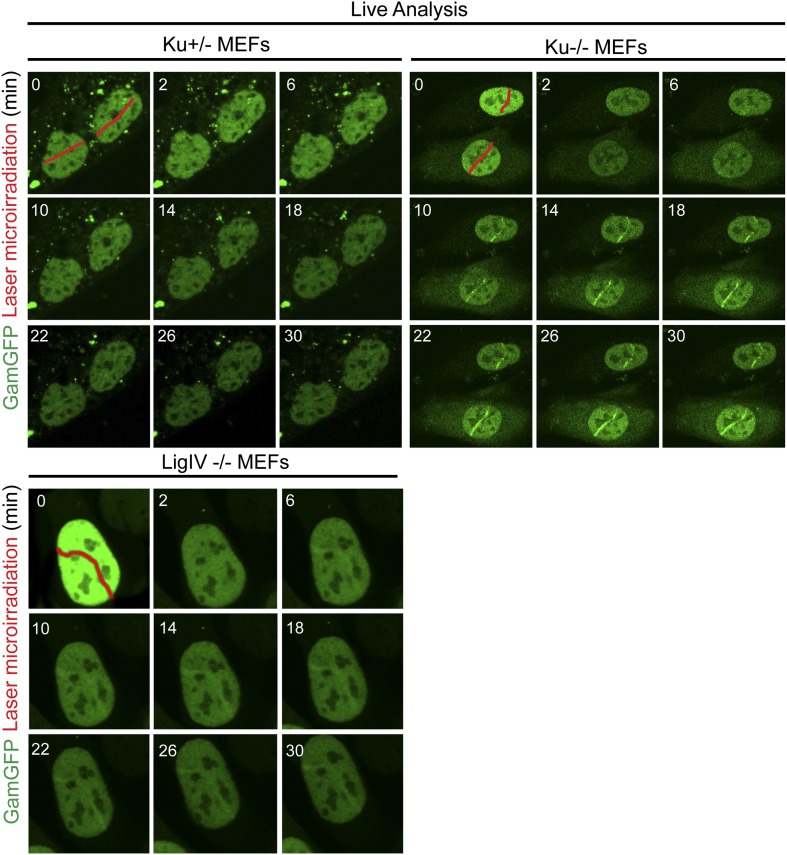
Ku inhibits GamGFP recruitment at DSBs independently of non-homologous end joining. Cells lacking either Ku80 or LigIV are defective in non-homologous end joining (NHEJ), yet the presence of Ku still inhibits recruitment of GamGFP to laser-induced DSBs even in NHEJ-defective cells, and thus independently of the cell's ability to complete NHEJ. Whereas it could have been possible that reduced GamGFP recruitment in the presence of Ku was caused by reduced persistence of DSBs due to their repair by NHEJ, our data show instead that Ku inhibits recruitment independently of successful NHEJ and imply that Ku binding to DSEs itself is inhibitory. **DOI:** http://dx.doi.org/10.7554/eLife.01222.014

### Incomplete 53BP1 co-localization with GamGFP

We examined co-localization of GamGFP with γ-H2AX and 53BP1 foci in Ku80-deficient MEFs, in which GamGFP binds DSBs efficiently, without inhibition by Ku (Figure 5B–D), as discussed above. We find that γ-H2AX labeling of laser damage co-localizes with GamGFP (Figure 5C). Interestingly, though both GamGFP and 53BP1 form foci on Gamma-irradiated Ku80-deficient MEFs, only ∼31% of foci per cell were coincident 53BP1 and GamGFP (Figure 5G). About 46% showed only 53BP1 and ∼23% showed only GamGFP (Figure 5G). The coincident foci of GamGFP with γ-H2AX (Figure 5C) and 53BP1 (Figure 5A,E–G) validate both of these markers, both indirect inferred DSB markers, as genuine DSB markers.

However, the data also imply that, as expected, some of the sites that 53BP1 binds might not, at the moment of binding, possess a frank DSE because 46% are not bound simultaneously by GamGFP (Figure 5G). This result is expected because, first, Gam is specific for flush DSEs with up to a four-base single-strand DNA overhang (Akroyd and Symonds, 1986), not the long single-strand DNA overhangs created by resection of DSBs by repair exonucleases (Symington and Gautier, 2011). Therefore, there are expected to be 53BP1 foci unoccupied by GamGFP, as we observe in Figure 5G, because GamGFP is a more specific reagent. Second, 53BP1 is expected to have a post-DSB-repair presence because it binds modified nucleosomes (Lukas et al., 2011b; Fradet-Turcotte et al., 2013) rather than DNA, which is also compatible with our data. The mechanism that predominates remains to be determined.

Similarly, the ∼23% of foci per cell with GamGFP alone indicates that some DSBs are present but not bound by sufficient 53BP1 to form a visible focus. These DSBs could lack DNA-damage-response signaling due to occlusion of the ends by GamGFP or exist in areas without the chromatin response that recruits 53BP1. At least in *E. coli*, GamGFP focus formation is very rapid, with 91 ± 0.5% (mean ± SEM, three independent experiments) of the foci resulting from a 10-min (pulse) exposure to 20 µg/ml phleomycin appearing at 10 min. Whether because of timing, location or signaling, the data suggest that a more accurate picture of the locations of current DSBs containing frank DSEs might be obtained with GamGFP than 53BP1.

### GamGFP inhibits IR-induced end resection

**Figure 6.**
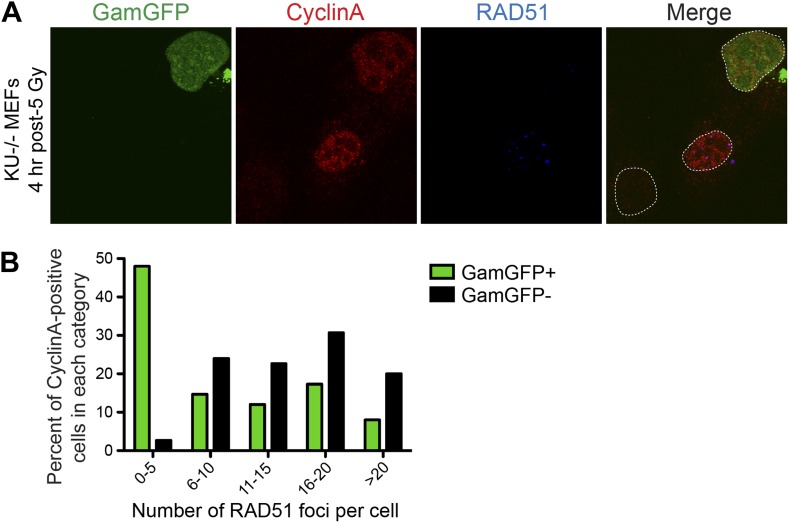
GamGFP inhibits IR-induced RAD51 foci, apparently blocking end resection. We quantified RAD51 foci (single-stranded DNA) (Raderschall et al., 1999) induced by IR, so presumably at DSBs, in S-G2 (CyclinA-positive) cells that either did or did not produce GamGFP, from the same transfections. (**A**) The GamGFP-positive Ku80-defective MEFs display reduced RAD51 foci upon IR treatment in S/G2 cells. Cells were analyzed by immunofluorescence with the indicated antibodies. S/G2 cells were identified by positive staining of CyclinA. Dotted white lines mark cell nuclei. (**B**) Quantification of RAD51 foci in CyclinA-positive cells with or without GamGFP production (cumulative values from three experiments with >75 cells total). Each cell is ‘Z-stacked’ (optically sectioned) so that all RAD51 foci were examined. The data indicate that most GamGFP-positive cells have few (0–5) RAD51 foci per cell, and that those cells with more RAD51 foci (classes 6–10, 11–15, 16–20 and >20) are enriched among the GamGFP-negative cells. These data indicate a partial mutual exclusivity of GamGFP presence and RAD51 foci, as would be expected if Gam binding to DSEs blocks the resection that creates the ssDNA onto which RAD51 binds. **DOI:** http://dx.doi.org/10.7554/eLife.01222.015

We find that GamGFP appears to block resection. We quantified RAD51 foci (single-stranded DNA) (Raderschall et al., 1999) induced by IR, thus including DSBs, in S-G2 (CyclinA-positive) cells that either were or were not simultaneously transfected with the GamGFP vector. We observed an inverse correlation between RAD51 foci and GamGFP-positive cells. Most of the cells that produced GamGFP had few (0–5) RAD51 foci, in optically sectioned nuclei in which all or most foci are expected to have been detected (Figure 6). Those nuclei without GamGFP had increased RAD51 focus formation. These data indicate exclusivity of the presence of GamGFP and resection, implying that as in *E. coli* (Figure 1C–F), GamGFP blocks exonuclease activity at DSEs in mammalian cells.

### APOBEC3A induces DSBs in human cells

**Figure 7.**
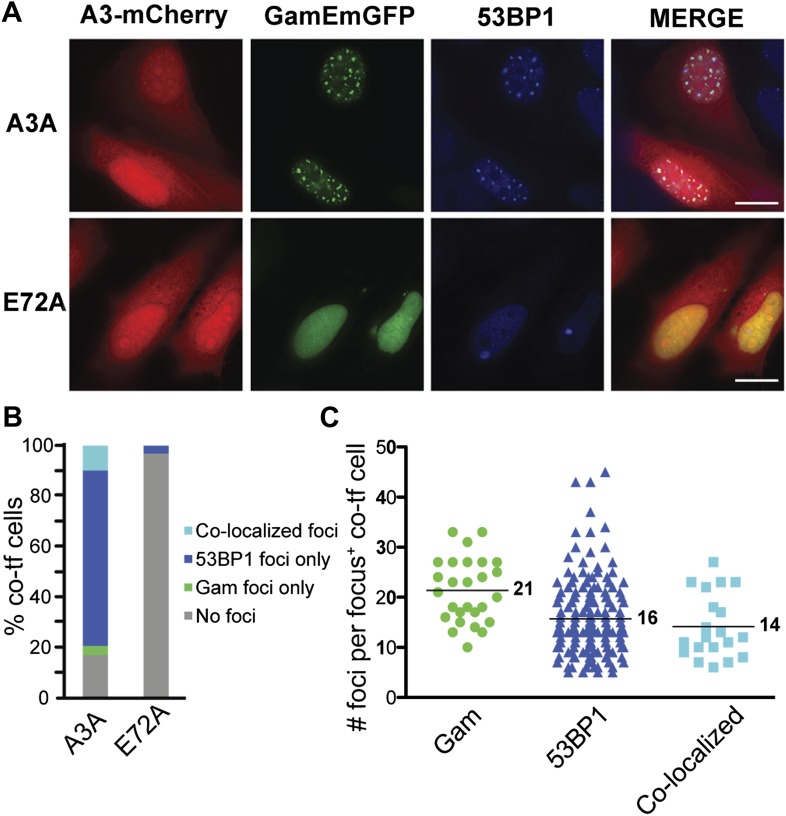
APOBEC3A induces DSBs in human cells. (**A**) HeLa cells co-transfected with GamEmGFP and APOBEC3A-mCherry or catalytic mutant, APOBEC3A-E72A-mCherry. (**B**) Summary of foci observed in cells producing both GamEmGFP and A3A-mCherry or A3A-E72A-mCherry (two independent experiments; n = 100 per experiment). Data are percent of co-transfected cells. (**C**) Mean number of foci per focus-positive cell co-transfected with and expressing both GamEmGFP and A3A-mCherry. **DOI:** http://dx.doi.org/10.7554/eLife.01222.016

APOBEC3A is one of the most potent members of a family of DNA cytosine deaminase enzymes (Carpenter et al., 2012). Induction of APOBEC3A, or the related enzyme APOBEC3B, results in both γ-H2AX and 53BP1 foci and frequent DNA breakage as determined by the comet assay (Landry et al., 2011; Shinohara et al., 2012; Taylor et al., 2013; Burns et al., 2013a). We expressed GamEmGFP (emerald GFP), one of the two forms we constructed in mammalian expression vectors. We find that GamEmGFP forms foci in ∼35% of cells when GamEmGFP and APOBEC3A are co-induced, and many of the GamEmGFP foci co-localize with 53BP1 (Figure 7). The appearance of foci requires the catalytic glutamate of ABOBEC3A, strongly implying that DNA cytosine deamination leads to DSBs in human cells. As in MEFs (Figure 5), incomplete localization of 53BP1 with GamEmGFP (Figure 7) implies that some 53BP1-bound sites may not have had Gam-recognizable DSBs, and some DSBs detectable by GamEmGFP did not possess sufficient 53BP1 to form a visible focus.

### Spontaneous DSBs in G1 phase

**Figure 8.**
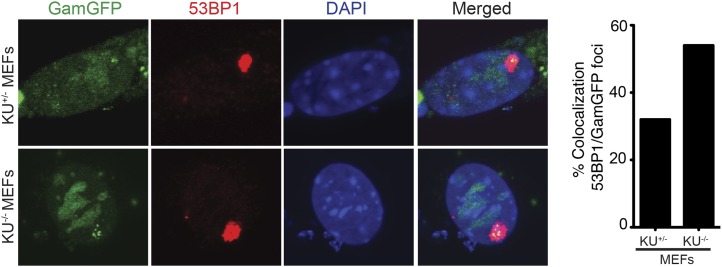
Spontaneous DNA breakage in G1-phase cells: GamGFP shows large spontaneous G1 53BP1 foci to contain multi-break clusters. The large spontaneous 53BP1 foci in undamaged cells, which occur solely in G1 (Harrigan et al., 2011*;* Lukas et al., 2011a), contain multiple DSBs that are marked by GamGFP. The GamGFP-53BP1 co-localization is more apparent in the absence of Ku. Data are from three (Ku80-proficient) or four (Ku80-defective) independent experiments of >25 cells each with Z-stacked (optically sectioned) nuclei. **DOI:** http://dx.doi.org/10.7554/eLife.01222.017

Most spontaneous DSBs are thought to occur during S-phase, presumably by any of several possible DNA replication-dependent mechanisms (reviewed in ‘Introduction’). However, 53BP1 forms unusually large nuclear foci exclusively in a subset of undamaged G1 cells (Harrigan et al., 2011; Lukas et al., 2011a). The large foci in otherwise undamaged cells are only in G1, as shown by their CyclinA-negative state and tracking fluorescent 53BP1 throughout the cell cycle (Harrigan et al., 2011; Lukas et al., 2011a). Although this might suggest that some spontaneous DSBs might form outside of S phase, it has been argued that not all 53BP1 foci indicate DSBs because different types of DNA damage nucleate 53BP1 foci (Lukas et al., 2011a). In this study, we examined the large nuclear 53BP1 foci in undamaged cells, previously shown to form exclusively in G1 (Harrigan et al., 2011; Lukas et al., 2011a), and find that ∼60% of the 53BP1 foci in Ku80-deficient cells correspond to genuine GamGFP-detectable DSBs (Figure 8). Moreover, the GamGFP that coincides with 53BP1 foci contain multiple individual foci, implying that these DSBs may be in large multi-break clusters. Because GamGFP does not spread along DNA, it can resolve multiple nearby DSBs, which was not possible with 53BP1 (Harrigan et al., 2011; Lukas et al., 2011a). These data indicate that some spontaneous DSBs form outside of S phase, when most replication occurs, and do so in clusters.

## Discussion

We showed that fluorescent-protein-fusion derivatives of the highly DSE-specific Gam protein of phage Mu allow direct identification of DSBs in bacterial and mammalian cells, and we used GamGFP to illuminate origins of spontaneous DSBs in both. In living *E. coli*, chromosomally encoded GamGFP forms foci at I-*Sce*I endonuclease-produced two-ended DSBs (Figures 2A–C and 3), at one-ended DSBs made by replication-fork collapse (Figure 2D,E), and DSBs generated by gamma-irradiation and bleomycin (Figure 2F, Figure 2—figure supplement 2). GamGFP detects about 71–82% of DSBs, a robust efficiency. A single GamGFP focus occurs per two-ended DSB (Figures 2A–C and 3) or per one-ended DSB (Figure 2E). Either *E. coli* DNA-repair or other proteins, or GamGFP itself, might keep the two ends in close enough proximity that only a single focus forms (discussed in ‘Results’: *GamGFP pinpoints subgenomic locations of DSBs in E. coli*). GamGFP also allowed subcellular localization of site-specific DSBs relative to a chromosomal mCherry marker in *E. coli*, which are distinct even as close as 55 kb (Figure 3). Similarly, GamGFP and GamEmGFP protein fusions produced in mammalian cells showed localization at laser-induced DSBs (Figure 5), were inhibited by Ku in those cells (Figure 5B–D), and inhibited resection at DSBs (Figure 6), indicating that GamGFPs label DSBs also in mammalian cells. GamGFP and its derivatives also allowed important novel conclusions about the origins of spontaneous DNA breaks in *E. coli* and mammalian cells.

In mammalian cells, GamGFPs may have considerable utility as a marker for DSBs, particularly when levels of DNA insults/damage are high. However, we note that a failure to detect DSBs using Gam may be due to competition with Ku (Figure 5B,C) and/or to relatively low levels of DNA damage. Additionally, in mammalian cells GamGFP foci were more dramatic in fixed than living cells (Figure 5A, Figure 5—figure supplement 1), perhaps because depletion of background unbound GamGFP in the fixed cells improved contrast. Nevertheless, this tool can be extremely valuable for a number of studies. For instance, GamGFPs allowed us to validate indirect markers γ-H2AX and 53BP1 as genuine DSB markers by showing co-localization at laser and IR-induced DSBs (Figures 5A,C,E–G, 7, 8), and GamGFP inhibits DNA end exonucleolytic resection (Figure 6). Consistent with these results, we also demonstrate that 53BP1 foci in G1 contain DSBs that previously had been inferred only (Figure 8). We are working to develop Ku-resistant DSE-binding fusions currently.

In work published while this paper was in review, Britton et al. (2013) use Ku foci for detection of DSBs similarly to our use of GamGFP. They show that Ku interacts with chromatin via RNA and that foci can be seen in RNase-treated samples. An advantage of Ku is that competition with Ku is not a problem, whereas an advantage of GamGFP is its greater specificity for double-strand ends (reviewed in ‘Introduction’).

Second, one aspect of the incomplete co-localization of 53BP1 with GamGFPs is that there are DSBs identifiable with GamGFPs that are not seen with 53BP1 (Figures 5G and 7C). One possible explanation is that, particularly in the Ku-defective MEFs (Figures 5G and 8), GamGFPs may be more sensitive than 53BP1 to DSBs, and might make visible DSB foci *before* DNA-damage signaling has allowed sufficient 53BP1 accumulation to produce a visible focus. At least in *E. coli* GamGFP labels DSBs very rapidly, showing most foci by 10 min after phleomycin exposure. Thus, in some circumstances, GamGFPs may be more sensitive than 53BP1. Conversely, the presence of 53BP1 foci at sites that do not show GamGFP foci (Figures 5E–G, 7B,C), even in Ku80-deficient MEFs (Figure 5D), may indicate that some of the sites bound by 53BP1 may not possess frank DSEs, the Gam DNA substrate (Williams and Radding, 1981). This could be because either the DSB has been repaired, but the 53BP1 at the site has not yet dissipated, or because exonucleolytic ssDNA resection has created a long ssDNA overhang, which cannot be bound by Gam (Williams and Radding, 1981; Akroyd and Symonds, 1986; Abraham and Symonds, 1990). With either possibility, the data suggest that GamGFPs may be more selective and specific markers for DSBs. Given the widespread use of 53BP1 as a DSB marker, understanding its potential limitations of possibly lower specificity relative to GamGFPs is important.

Third, we demonstrate directly that primate-specific deaminase APOBEC3A caused GamGFP foci, and thus DSBs in human cells, indicating that DSBs result from DNA cytosine deamination. This supports previous, less specific evidence from the appearance of γ-H2AX (Landry et al., 2011; Burns et al., 2013a) and 53BP1 (Taylor et al., 2013) foci induced by APOBEC3A expression. A possible mechanism could be that removal of uracil from DNA after cytosine deamination followed by cleavage at the abasic site creates a ssDNA nick, which becomes a one-ended DSB either by replication-fork collapse (Figure 2D), or similar ssDNA nick creation on the opposing strand. DNA breaks occur upon uracil excision from DNA in *E. coli* (Kuzminova and Kuzminov, 2008). Regardless of the specific mechanisms, the data indicate that cytosine-deamination may be a general mechanism of DSB generation in mammalian cells. Cytosine deamination clearly contributes a significant part of the cancer genome mutational landscape (Nik-Zainal et al., 2012; Roberts et al., 2012; Taylor et al., 2013; Burns et al., 2013a, 2013b) and it is likely that some of this could be by DSB induction.

In *E. coli*, GamGFP allowed demonstration that, first and importantly, spontaneous DNA breakage is precisely correlated with the number of cell divisions (Figure 4), providing the first evidence that most spontaneous breakage results from DNA replication-based mechanisms. Previously, DNA replication has been implicated in several mechanisms of DSB formation. These include fork collapse in engineered lambda phage (Kuzminov, 2001), fork-regression or ‘chicken-foot’ formation, to produce one-ended DSBs, in cells with defective replication proteins (Michel et al., 1997), and DSBs created by collisions of replication forks with transcription complexes (Merrikh et al., 2012), and other proteins (Gupta et al., 2013). However, nearly all of the work required engineered constructs or situations (to maximize collisions, fork collapses, etc) or mutant proteins, and so did not address how *spontaneous* DSBs occur normally. Our results demonstrate the correspondence of spontaneous DSBs with the number of cell divisions and imply that some or all of these mechanisms, and replication generally, are in fact predominant causes of spontaneous DNA breakage normally in proliferating *E. coli.*

In separate work, we found that one particular replicative mechanism, in which RNA (R-loop)-primed replication-fork collapse produces DSBs, occurs spontaneously in stationary-phase (non-proliferating) *E. coli* (Wimberly et al., 2013).

Second, the demonstration that spontaneous DSBs are generation dependent (Figure 4A), and our finding that most spontaneous DSBs result as single events per cell, not multi-break catastrophes (Figures 2C and 4, Figure 4—figure supplement 1), allowed calculation of the first true rates of spontaneous DNA breakage. Our microfluidic data directly show rates of 0.021±0.008 spontaneous DSBs per cell division, and the use of our data to re-interpret and correct previous indirect, low-resolution data (Pennington and Rosenberg, 2007) indicates a roughly similar rate of 0.011 spontaneous DSBs per cell division (‘Results’). These true rates are 10–20-times lower than initial postulates (Cox et al., 2000), but in line with each other. Because genomic rearrangement frequencies remain the same regardless of estimations of DSB numbers, these rarer and more accurate break rates imply that each DSB is 10–20 times more genome destabilizing than had initially been postulated. Spontaneous DSBs are infrequent but dangerous, and now that their generation-dependence is unequivocal, their origins in replication are supported.

Whereas mechanisms underlying spontaneous DNA breakage have been elusive, our data support major roles for replication in *E. coli*, and for some DNA breakage outside of S phase, in G1 in human cells. Our results show a novel DNA breakage mechanism in human cells, and indicate the utility of fluorescent-Gam for interrogating DSB formation, location, and dynamics in living cells. Understanding the origins of spontaneous DNA breakage obtainable with improved synthetic reagents such as GamGFP will illuminate the underlying causes, and possible means of prevention, of important biological consequences of DNA breakage in living cells.

## Materials and methods

### Strains, media and growth

*E. coli* strains used are given in Supplementary file 1A. Bacteria were grown in LBH (Torkelson et al., 1997) or M9 minimal medium (Miller, 1992) supplemented with 10 μg/ml thiamine (vitamin B1) and 0.1% glucose as carbon source. Other additives were used at the following concentrations (μg/ml): ampicillin, 100; chloramphenicol, 25; kanamycin, 50; tetracycline, 10; gentamycin, 15; sodium citrate 20 mM. Human HeLa cells were grown in Dulbecco’s modified Eagle’s medium (DMEM) supplemented with 10% fetal bovine serum (FBS), 100 µg/ml penicillin, 100 mg/ml streptomycin and 2 mM L-glutamine. SV40 immortalized MEF cells lines (Ku80-defective *Xrcc5*^+/−^, Ku80-proficient *Xrcc5*^*−/−*^*, Lig4*^*−/−*^; kindly provided by the Lazzerini Denchi lab, Scripps Research Institute*)* were grown in the same medium containing 15% FBS.

### Cloning and chromosomal expression cassettes encoding Gam and GamGFP in *E. coli*

#### Chromosomal doxycycline-inducible expression cassette

The bacteriophage Mu *gam* gene from plasmid pJA21 (Akroyd et al., 1986) was purified after *Eco*RI restriction enzyme digestion and subcloned into the *Eco*RI site of plasmid pRF3 (this study) downstream of the doxycylcine-inducible P_N25*tetO*_ promoter (Nishihara et al., 1998). The plasmid pRF3 was constructed from pRF1 (this study) by linking the constitutive P_N25_ promoter sequence (Nishihara et al., 1998) with a chloramphenicol resistance (*cat*) cassette flanked by FRT sites, removed as a *Hin*dIII fragment from plasmid pKD3 (Datsenko and Wanner, 2000) and inserted into the pRF1 *Hin*dIII site. This makes the promoter-*cat* pair easily transferrable to the chromosome. The promoter P_N25_ sequence was amplified from the plasmid pGro11 (Nishihara et al., 1998) using primer P1 and P2 (Supplementary file 1B) and subcloned into the *Eco*RI/*Sac*I sites of construction vector pMOD5 from Epicentre to create pRF1.

P_N25*tetO*_ can be induced by tetracycline or doxycycline. The *tetR* gene, which is required to repress genes under control of P_N25*tetO*_, was cloned downstream of a constitutive P_N25_ promoter (Nishihara et al., 1998) to create P_N25_*tetR*. Part of the *lacI*^*q*^ allele next to the P_N25_*tetR* fusion of the original construction was replaced with FRTKanFRT from pKD4 using the primers P3 and P4 (Supplementary file 1B). Subsequently, P_N25_*tetR* was placed downstream of *yfeP* in the *E. coli* chromosome by phage λ Red recombineering using primers P5 and P6 (Supplementary file 1B). In the absence of doxycycline or tetracycline (inducer), the constitutively produced TetR protein represses the P_N25*tetO*_ promoter, keeping *gam* and *gam-gfp* tightly off in *E. coli*. Upon addition of doxycycline, TetR undergoes a conformational change causing release from *tetO*, relieving repression, and allowing transcription of the downstream gene. P_N25*tetO*_*gam* from the plasmid pRF3gam was transferred into the *E. coli* chromosome at the Tn*7* attachment (*attTn7*) site by Red short-homology recombineering using primers P7 and P8 (Supplementary file 1B). Similarly, a GamGFP fusion was constructed by placing P_N25*tetO*_*gam* just upstream of the *gfp* coding region in strain SMR13957, linked with six alanines using primers P9 and P10 (Supplementary file 1B) at *attTn7*. Promoter-only and GFP-only control strains were constructed by Red short homology recombineering into *attTn7* in the *E. coli* chromosome using primer P11–P12 and P13–P14 respectively (Supplementary file 1B).

#### Chromosomal temperature-inducible expression cassette

For experiments assaying GamGFP co-localization with TetR-mCherry on a chromosomal *tetO* array (Figure 3), we required and constructed a TetR-independent expression cassette for *gam-gfp*. We cloned *gam-gfp* (above) under the control of the phage λ P_R_ promoter, next to the λ*c*I*ts857* gene, encoding a temperature-sensitive CI transcriptional repressor protein that represses λ P_R_ at ≤34°C and allows transcription from λ P_R_ at ≥37°C. We first linked a FRT (FLP-recombinase site)-flanked chloramphenicol resistance (*cat*) cassette from plasmid pKD3 with *c*I*ts857* and the nearby λ P_R_ promoter in the chromosome of *E. coli* λ-lysogen strain SMR16402. The newly constructed control region was placed upstream of *gam-gfp* in the *E. coli* chromosome in *attTn7* to create *attTn7*::FRT*cat*FRT λ*c*I*ts857* P_R_*gam-gfp*. At permissive temperature (≤34°C) the CI repressor protein binds P_R_ and represses transcription of P_R_*gam-gfp*. At ≥37°C, the CI repressor breaks and GamGFP is produced.

### Cloning and production of GamGFP and GamEmGFP for mammalian cells

*gam*-*gfp* was amplified from *E. coli* SMR14354 genomic DNA with 5′-NNN NAA GCT TGC CAC CAT GGC TAA ACC AGC AAA ACG-3′ and 5′-GCA GCC GGA TCC CTT ATT TGT ATA GTT C-3′, digested with *Hin*dIII and *Bam*HI, and ligated into similarly cut pcDNA3.1(+) (Invitrogen, Carlsbad, CA, USA). *gam-EmGFP* was constructed by first amplifying *gam* from pcDNA3.1(+)-Gam-EGFP using 5′-NNN NAA GCT TGC CAC CAT GGC TAA ACC AGC AAA ACG-3′ and 5′-CCT CGC CCT TGC TCA CCA TAT GTA TAT CGG CGG CGG CGG C-3′. Second, *EmGFP* was amplified from plasmid pcDNA6.2-C-EmGFP-DEST (Invitrogen, Carlsbad, CA, USA) with 5′-GCC GCC GCC GCC GAT ATA CAT ATG GTG AGC AAG GGC GAG G-3′ and 5′-NNN NGG ATC CTT ACT TGT ACA GCT CGT CCA TGC CG-3′. Finally, these two fragments were used in an overlapping PCR with the 5′-Gam and 3′-EmGFP primers above to make a *gam-EmGFP* fusion gene, which was digested with *Bam*HI and *Hin*dIII, and ligated into similarly cut pcDNA3.1(+) (Invitrogen, Carlsbad, CA, USA). The constructs were verified by restriction digestion and DNA sequencing prior to functional assays.

### Expression and microscopy of Gam-EmGFP in mammalian cells

The APOBEC3A-mCherry construct and the catalytic mutant construct, APOBEC3A-E72A-mCherry, were previously described (Lackey et al., 2012). HeLa cells at 75% confluence were co-transfected transiently with 150 ng of *gam-EmGFP* and APOBEC3A-mCherry or APOBEC3A-E72A-mCherry constructs using TransIt-LT1 (Mirus, Madison, WI, USA) and fixed 20 hr later in 4% paraformaldehyde. 53BP1 foci were detected by rabbit anti-53BP1 (1:1000; Novus Biologicals, Littleton, CO, USA) followed by goat anti-rabbit Cy5 (1:500; Abcam, UK). Nuclei were stained with 0.1% Hoechst dye for imaging at 600X magnification using a DeltaVision deconvolution microscope (Applied Precision, Issaquah, WA, USA).

### SDS-PAGE and western blot

Production of Gam and GamGFP in *E. coli* was determined by SDS-PAGE and western blot analysis. Saturated LBH cultures grown at 37°C were diluted 1:100 in a fresh medium and grown 4 hr with or without addition of 200 ng/ml doxycycline. Total proteins were extracted from 1-ml culture with BugBuster^®^ master Mix extraction buffer (Novagen^®^, Madison, WI, USA). Samples were denatured with 6X SDS loading dye, heated to 95°C for 5 min and 10 μl of total proteins were loaded onto a 12% polyacrylamide gel. Sodium dodecyl sulfate-polyacrylamide gel (12%) electrophoresis (SDS-PAGE) under reducing conditions was performed as per Laemmli (1970). Relative molecular weights were determined using Precision Plus Protein Kaleidoscope Standards. Proteins were detected by staining the gel with 0.1% Coomassie brilliant blue R-250.

For western blot analysis of GFP and GamGFP, gels were transferred to PVDF membrane according to manufacturer’s specifications (Amersham, GE Healthcare, Piscataway, NJ, USA). The membranes were blocked with 5% non-fat dry milk in PBS-Tween and probed with primary rabbit anti-GFP polyclonal IgG antibodies from Santa Cruz Biotechnology (Santa Cruz, CA, USA). Bound antibodies were detected using secondary antibody ECL Plex goat-a-rabbit IgG conjugated with fluorescent dye Cy5 after 1000-fold dilution and visualized by scanning in multicolor imager Typhoon detection system.

### Phage lambda *red gam* plaque assay for Mu Gam function

Functional validation of Mu Gam and GamGFP produced in *E. coli* was determined by a lambda (λ) plaque assay. A λ*red gam* Chi^0^ strain was used to test the activity of Mu Gam and GamGFP in *E. coli.* Saturated tryptone broth (TB) cultures of *E. coli* were diluted 1:10 in fresh TB with 0.2% maltose, 5 mM MgSO_4_, 10 μg/ml thymine, 10 μg/ml vitamin B1 and grown for half an hour prior to the addition of 200 ng/ml of doxycycline to induce production of Gam and GamGFP. After 2 hr of induction by doxycycline shaking at 37°C, an equal volume of 10 mM Tris 10 mM Mg (TM) buffer, pH 7.5 was added and cells were mixed with an appropriate volume of λ*red gam* Chi^0^ suspension, adsorbed without shaking for 10 min at room temperature, then plated with the addition of 2.5 ml molten 50°C soft BBL agar (1% BBL trypticase peptone, 0.1M NaCl, 0.7% agar, pH 7.5) onto BBL plates (same medium solidified with 1% agar) with or without 200 ng/ml doxycycline. The plates were incubated overnight at 37°C before scoring size of plaques.

### UV-sensitivity test

Saturated LBH cultures of *E. coli* were diluted 1:100 in fresh LBH medium and grown shaking at 37°C for 90 min, at which time 200 ng/ml of doxycycline was added to induce Gam and GamGFP production. After 2 hr of induction by doxycycline at 37°C shaking, cells were plated on LBH solid medium containing 200 ng/ml doxycycline and the plated cells irradiated with different UV doses, then incubated in the dark over night at 37°C for colony quantification. Control cultures without doxycycline induction were treated otherwise identically.

### Spontaneous and I-*Sce*I–induced foci in *E. coli*

Saturated overnight cultures of *E. coli* carrying the chromosomal *gam-gfp* expression cassette were diluted 1:100 in fresh medium, 100 ng/ml doxycycline added to induce GamGFP, incubated for 1 hr shaking at 37°C, then 0.1% arabinose added to induce I-*Sce*I production and incubated 3 hr more, then subjected to microscopic examination. Live cells were visualized using a Zeiss inverted fluorescence microscope with Axion vision software. Quantification of foci was done by Nick software.

### Quantitative PCR

Genomic DNA was prepared from *E. coli* incubated as described above for the I-*Sce*I induced DSB-associated GamGFP focus experiments using a CTAB preparation protocol (Porebski et al., 1997). Reactions containing 10 ng genomic DNA and 350 µM primers P21 (*oriC* specific) or P22 (*terC* specific) in 1X KAPA SYBR Fast ABI prism qPCR mix (20 µl total volume) were run in an Applied Biosystems (Foster City, CA, USA) 7900HT RT thermocycler in 96-well plates. Relative copy numbers of *oriC* and *terC* sequences were determined by the ΔCt method: the difference in amplification rates of *oriC* and *terC* (ΔCt) for each sample (average of four replicate reactions) were normalized to the ΔCt for a wild-type strain grown to saturation (stationary phase) shaking in LBH at 37°C for 28 hr, to contain equal numbers of *oriC* and *terC* sequences per cell.

### DNA damaging agents and GamGFP foci

The saturated cultures of *E. coli* grown in LBH (for bleomycin experiments) or M9 0.4% glucose (for gamma irradiation) medium were diluted 1:100 into fresh LBH or M9 0.4% glucose, then grown 1 hr shaking at 37°C, then given 100 ng/ml doxycycline to induce GamGFP, grown an additional 1 hr, and treated with 20 µg/ml of bleomycin, or different doses of gamma radiation. A Cs^137^ or a Faxitron X-ray machine (Faxitron X-ray Corporation, Tucson, AZ, USA) was used for gamma irradiation (experiments in Figures 2 and 5, respectively). After treatment with DNA-damaging agents or irradiation, cells were incubated for an additional 3 hr before microscopic examination unless stated otherwise. Gamma irradiation was done in exponential cultures grown in M9 0.4% glucose with shaking at 37°C to mimic the identical growth medium and growth conditions used previously for quantification of DSBs/Gy IR in *E. coli* by Bonura and Smith (1977). Elsewhere in the paper, cultures are grown in M9 vitamin B1 0.1% glucose, or LBH, and gave different numbers of foci per cell (more in LBH, less in M9 B1 0.1% glucose) in these richer and poorer growth media.

### Co-localization of GamGFP and TetR-mCherry

TetR mCherry was expressed from the arabinose inducible P_*BAD*_ promoter in plasmid pDB340 in cells induced for GamGFP production at 37°C, at which temperature the chromosomal λ*c*I*ts857-*controlled P_R_*gam-gfp* cassette is transcribed. Estimation of average inter-focal distances and co-localization of GamGFP and TetR-mCherry was determined from cells that contained both green and red foci at a 1:1 ratio. In the case of cells with DSBs near *ter,* many cells had two red foci and one green focus. In this case, interfocal distances are plotted separately for cells that contained either a 1:1 or 1:2 ratio of green and red foci (Figure 3K, 2.4 Mb). In the case of two red foci, the longer and shorter interfocal distances between the red and green foci are plotted separately as ‘1:2 far’ and ‘1:2 near’, respectively (Figure 3K). Multicolor fluorescent beads on slides were used to align independent color channels. All images were acquired with a Zeiss Axio Imager Z1 microscope plus Hamamatsu Electron Multiplier charge-coupled device (CCD) camera.

### Microfluidics and time-lapse fluorescence microscopy of *E. coli*

We followed the growth of single cells into microcolonies using the CellASIC ONIX Microfluidic Platform (Millipore, Billerica, MA, USA) including microfluidic perfusion system, microfluidic flow chamber for bacteria (BO4A plates) and FG software. All experiments were performed at 2psi (flow rate of 3 µl/hr). Time-lapse microscopy was performed using a Zeiss HAL100 inverted fluorescence microscope. Fields were acquired at 100× magnification with an EM-CCD camera (Hammamatsu, Japan). Bright field and fluorescence images (GFP cube = Chroma, #41017; X-Cite120 fluorescence illuminator, EXFO Photonic Solutions) were acquired and image analysis performed using AxioVision Rel. 4.6 (Zeiss, Germany). The microscope was housed in an incubation system consisting of Incubator XL-S1 (PeCon, Germany) controlled by TempModule S and Heating Unit XL S (Zeiss, Germany) to maintain a constant 37°C environment throughout the experiments.

#### Growth protocol

In these experiments, saturated cultures of SMR14350 were diluted 10,000-fold in M9 B1 0.1% glucose medium, grown to log phase shaking at 37°C, then GamGFP was induced by addition of 10 ng/ml doxycycline for 2 hr during shaking growth at 37°C prior to loading cells into the microfluidics chamber (time 0). For the next 9 hr, cells were bathed with M9 B1 glucose doxycylcine medium to allow division, then switched at 9 hr to the same medium without glucose, and bathed for an additional 18 hr. The number of cell divisions and the appearance of GamGFP foci were recorded using time-lapse microscopic photography throughout the experiment. After 27 hr, cells were treated with 20 μg/ml phleomycin to induce DSBs, to determine whether GamGFP foci could still form in the starved cells. We observed that 45 ± 5% of the non-dividing cells produced GamGFP at that time, confirming that the failure to see GamGFP foci in the cells starved prior to that time reflects lack of spontaneous DSBs, not inability of foci to form if their had been DSBs. The data presented in Figure 4A show the means ± SEM of results from six individual cells that grew into microcolonies in the microfluidic chamber.

#### Evidence that fluorescence exposure did not contribute to the spontaneous GamGFP foci scored

Because repeated exposures of samples to high doses of exitatory fluorescent light in time-lapse photomicroscopy has the potential to cause DNA damage (Ge et al., 2013), which might appear as foci at later time points, we used a low-dose of excitatory light for fluorescence with 30 ms at each exposure. Moreover, we show experimentally that the repeated low-dose exposures of the developing microcolonies did not cause an increase in GamGFP foci by scoring control experiments in which a set of six developing microcolonies were not exposed to the first 9 pulses of fluorescent light, during the initial 27 hr of growth, then were photographed for the first time at 27 hr, which corresponds the to tenth light pulse in experimental samples scored in Figure 4A. These previously unexposed samples showed 0.018 ± 0.005 foci per cell at 27 hr, a number that is not significantly different (p*=*0.611, student’s *t* test) from the repeatedly pulsed experimental samples at 27 hr, which showed 0.016 ± 0.002 foci per cell at 9 hr. These data demonstrate that the spontaneous DNA breaks measured in the microfludics experiments were not significantly augmented with DNA breaks induced by repeated fluorescence imaging, and rather reflect genuine spontaneous DSBs.

### Laser microscopy of GamGFP foci in mammalian cells

For laser-induced damage, cells were grown on glass-bottomed dishes (Willco Wells, Netherlands) and pre-sensitized by adding 1.5 µM BrdU (5-bromo-2′-deoxyuridine) for >20 hr followed by laser micro-irradiation. DNA damage was created using a 405 nm solid-state laser focused through a 63X objective lens in the epifluorescence path of the microscope system on a Fluoview 1000 confocal microscope (Olympus, Japan). Laser settings (60% laser power, 150 scans at 20 ms/pixel) were used that generated DNA damage specifically along the laser path in a BrdU-dependent manner. The cells were analyzed in TimeScan mode using Zero-drift compensation for auto focusing at an interval of 1–2 min for up to 30 min post-laser damage for live analysis. Images were exported as TIFF files and analyzed in ImageJ.

### Immunofluorescence in mammalian cells

After DNA damage induction, the cells were washed three times with cold 1× PBS, then pre-extracted by incubating the dishes in CSK buffer (10 mM PIPES pH 6.8, 100 mM NaCl, 300 mM sucrose, 3 mM MgCl_2_, 1 mM EGTA, 0.5% TritonX-100) for 1 to 5 min on ice. Cells were washed three times in room-temperature-PBS and fixed with 2% paraformaldehyde for 15 min at room temperature followed by three washes with 1× PBS, then blocked with 3%BSA in PBS for 15 min, and incubated with either 53BP1 (Novus, Littleton, CO, USA, NB100-304) or γ-H2AX (Millipore, Billerica, MA, USA, #05-636) for 1 hr at room temperature, or overnight at 4°C. The cells were washed three times in PBS followed by a 45 min incubation in PBS containing Alexa Fluor 594 or 647 goat-anti rabbit IgG to detect 53BP1 or Alexa Fluor 594 goat-anti mouse IgG to detect γ-H2AX. Hoechst dye (0.1%) was added to this incubation step to stain nuclear DNA, followed by washing three times in PBS and mounting on coverslips containing Vectashield. The cells were imaged using an inverted Fluoview 1000 confocal microscope (Olympus, Japan). To quantify laser recruitment of GamGFP, the cells were laser damaged and analyzed by immunofluorescence as described. Images from individual cells were taken and equal areas both in undamaged and damaged areas (as indicated by 53BP1 positive staining) were quantified for Gam signal using Fluoview software. The cells that showed a >30% increase in intensity of GamGFP at the damage site compared with the undamaged site were labeled positive. This analysis was performed in both Ku80-proficient and Ku80-defective MEFs for >50 cells and results are means ± SEM of three independent experiments. For quantification of GamGFP IR foci, cells were damaged and analyzed by immunofluorescence as indicated. Individual images were taken and foci counted that either contained GamGFP only, 53BP1 only, or both. The average total number of foci per cell 30 min post-5Gy IR was 34. For >2600 foci counted, the mean percentage of foci for each of the three categories is graphed for three independent experiments ± SEM.

For quantification of RAD51 foci in CyclinA-positive cells, Ku80-defective MEFs were transfected with GamGFP, treated with IR and analyzed 4 hr post-5 Gy IR. The cells were analyzed by immunofluorescence as described above except without pre-extraction with CSK buffer. RAD51 (Abcam, ab88572) and CyclinA (Santa Cruz, Santa Cruz, CA, USA, sc-751) antibodies were applied for 1 hr room temperature. AlexaFluor 594 goat anti-mouse and AlexaFluor 647 goat anti-rabbit IgG were used to fluorescently label RAD51 and CyclinA respectively and the cells were fixed as described. Z-stacked images were collected using a Fluoview FV1000 confocal microscope and RAD51 foci were counted in CyclinA-positive cells with or without GamGFP signal. Data from three independent experiments from >75 cells are graphed (Figure 6).

For detection of spontaneous/endogenous G1 foci, we followed the demonstration of Harrigan et al. (2011); Lukas et al. (2011a) that large 53BP1 foci in undamaged cells occur almost exclusively in G1 phase, as shown by their CyclinA-negative state and tracking of fluorescent 53BP1 throughout the cell cycle. We, therefore, used the criterion of large spontaneous 53BP1 foci to identify G1 cells.

## Additional files

Supplementary file 1. (**A**) *Escherichia coli* K12 plasmids and strains used in this study. *E. coli* strains and plasmids were constructed using standard P1 transduction (Miller, 1992), transformation, lysogenization (Gumbiner-Russo et al., 2001), recombinant DNA (Sambrook and Russell, 2001) and phage λ Red-mediated short-homology recombineering methods (Datsenko and Wanner, 2000). Sequencing was performed by SeqWright DNA Technology Services (Houston, TX). (**B**) PCR primers. **DOI:** http://dx.doi.org/10.7554/eLife.01222.018
